# Improving medication adherence monitoring and clinical outcomes through mHealth: A randomized controlled trial protocol in pediatric stem cell transplant

**DOI:** 10.1371/journal.pone.0289987

**Published:** 2023-08-17

**Authors:** Jessica E. Ralph, Emre Sezgin, Charis J. Stanek, Wendy Landier, Ahna L. H. Pai, Cynthia A. Gerhardt, Micah A. Skeens

**Affiliations:** 1 The Abigail Wexner Research Institute at Nationwide Children’s Hospital, Columbus, Ohio, United States of America; 2 The Ohio State University College of Medicine, Columbus, Ohio, United States of America; 3 University of Alabama Birmingham School of Medicine, Birmingham, Alabama, United States of America; 4 Cincinnati Children’s Hospital Medical Center & University of Cincinnati, Cincinnati, Ohio, United States of America; UNITED KINGDOM

## Abstract

Medication non-adherence rates in children range between 50% and 80% in the United States. Due to multifaceted outpatient routines, children receiving hematopoietic stem cell transplant (HCT) are at especially high risk of non-adherence, which can be life-threatening. Although digital health interventions have been effective in improving non-adherence in many pediatric conditions, limited research has examined their benefits among families of children receiving HCT. To address this gap, we created the BMT4me© mobile health app, an innovative intervention serving as a “virtual assistant” to send medication-taking reminders for caregivers and to track, in real-time, the child’s medication taking, barriers to missed doses, symptoms or side effects, and other notes regarding their child’s treatment. In this randomized controlled trial, caregivers will be randomized to either the control (standard of care) group or the intervention (BMT4me© app) group at initial discharge post-HCT. Both groups will receive an electronic adherence monitoring device (i.e., medication event monitoring system “MEMS” cap, Medy Remote Patient Management “MedyRPM” medication adherence box) to store their child’s immunosuppressant medication. Caregivers who agree to participate will be asked to complete enrollment, weekly, and monthly parent-proxy measures of their child’s medication adherence until the child reaches Day 100 or complete taper from immunosuppression. Caregivers will also participate in a 15 to 30-minute exit interview at the conclusion of the study. Descriptive statistics and correlations will be used to assess phone activity and use behavior over time. Independent samples t-tests will examine the efficacy of the intervention to improve adherence monitoring and reduce readmission rates. The primary expected outcome of this study is that the BMT4me© app will improve the real-time monitoring and medication adherence in children receiving hematopoietic stem cell transplant following discharge, thus improving clinical outcomes.

## Introduction

Poor adherence accounts for up to 70% of all medication-related hospital admissions [1, 2] in the United States, resulting in approximately $100 billion in healthcare costs annually [3]. Non-adherence rates have been reported as high as 50–80% in pediatric chronic illness populations [4–6]. and 51 to 66% in adult hematopoietic stem cell transplant (HCT) patients [7, 8]. A growing body of evidence in chronic illnesses suggestst non-adherence is associated with adverse clinical outcomes, including infectious complications, hospital admissions, and even death [9–12]. However, the impact of non-adherence on pediatric HCT clinical outcomes is largely unknown.

Hematopoietic stem cell transplant patients are most immunocompromised during the first 100 days [13], thus during this time, HCT recipients must adhere to multifaceted and complex outpatient medication regimens. Specifically, adherence to immunosuppressant medications during the acute phase (first 100 days) post-transplant is critical to prevent graft versus host disease (GVHD) and avoid graft failure [7, 14, 15]. Children that develop acute GVHD have a 30% to 50% chance of survival. Morbidity and mortality due to GVHD can be decreased through prophylactic use of immunosuppressants [14, 16, 17]. Although these medications are costly and produce unpleasant side effects [14], adherence is critical to decrease complications, reduce readmissions, and ultimately increase quality of life and survival [10].

The complexity and duration of treatment regimens, along with forgetfulness, have been consistently identified as the primary determinants of medication non-adherence [6, 10, 11, 18–20]. Specifically, reasons for pediatric non-adherence are multifactorial and encompass various factors [20–22] As primary caregivers, they are responsible for filling prescriptions, retrieving medications, and ensuring their child receives the prescribed therapy correctly [23–25]. In the high-risk HCT population, caregivers are isolated with their child due to infection risk and must manage challenging treatment regimens at home, often with limited time and support. Complex behavioral interventions, typically employed to address non-adherence, are difficult to deliver and manage in the context of these daily tasks, especially when one considers the geographic, resource, and time constraints families of chronically ill children face [26].

mHealth apps offer solutions to address non-adherence, reduce potential barriers, and are widely available, simple, and innovative [27–32]. However, their feasibility, acceptability, and usability, especially in pediatric care, remain uncertain. Recent reviews of pediatric medical adherence apps found that none identified individual barriers to adherence [33] and, specific to the pediatric oncology population, nearly all were designed solely for education or nonmedication-related purposes [34]. Similarly, to the best of our knowledge, limited applications and research exist regarding adherence in pediatric HCT, and broad gaps exist with regard to adherence to immunosuppressant medication.

To address these gaps, this longitudinal, pilot RCT protocol reports the design and methods of a preliminary acceptability and efficacy study of the BMT4me© mobile health app (referred to hereinafter as the “BMT4me© app”), as well as the feasibility of enrolling and retaining 50 caregivers of children during the acute, outpatient phase (i.e., first 100 days) post-HCT.

### Hypotheses

We hypothesize caregivers enrolled in this study will: (a) report above average acceptability (≥ 68%) of the BMT4me*©* app, and ≥ 75% of participants will enroll and complete all study-related assessments in both arms, (b) have higher adherence frequency, if randomized to the BMT4me*©* app, than the standard of care group, and (c) have less GVHD and fewer readmissions than the standard of care group.

## Methods

### Design

This is a longitudinal pilot randomized controlled trial (RCT) designed to test the efficacy of the BMT4me© app intervention on adherence to immunosuppressant medication in families of children discharged during the acute phase post-HCT. This study will take place at Nationwide Children’s Hospital (NCH), a large, academic, free-standing pediatric hospital in the Midwestern United States. Children and primary caregivers will be screened for eligibility during HCT, and caregivers of eligible participants will be approached regarding the study prior to the child’s discharge from the hospital. Participating families will be randomized (1:1) to either the standard of care control group or the BMT4me© app intervention group. Families in both groups will be assigned medication adherence monitoring devices and complete follow-up assessments once per week during the acute phase (i.e., approximately 11 weeks or 100 days) or until the child completes their immunosuppression medication taper. The objective of this RCT is to evaluate the acceptability of the newly developed BMT4me© app and the feasibility of enrolling and retaining 50 caregivers of children in the acute phase post-HCT. The secondary objective is to evaluate the potential efficacy of the BMT4me© app on adherence to immunosuppressant medications in children who have been discharged home during the acute phase post-HCT.

### Setting and participants

Participants will include primary caregivers of children post HCT. Primary caregivers will be identified by study staff through NCH’s HCT unit electronic medical record prior to the child’s discharge. Children of caregivers must be: (a) 0 to 21 years of age, (b) receiving immunosuppression for an allogenic transplant, (c) discharged prior to day 100 or immunosuppression taper, and (d) residing with a primary caregiver that enrolls on the study. Primary caregivers must be: (a) English-speaking and (b) have an iOS or Android capable cellular device. To be inclusive of varying family structures, eligible primary caregivers may also include any legal guardian of the child (e.g., adoptive parent, grandparent, aunt/uncle, etc.). Caregivers are ineligible if they are unable to consent or the participating child has a documented developmental delay.

### Sample size calculations

The overall goal of this pilot RCT is to examine preliminary acceptability and efficacy of the BMT4me© app and assess the feasibility of enrolling and retaining 50 caregivers of children in the acute phase post-HCT. The sample of 50 caregivers (25 intervention, 25 standard of care) was chosen to inform a multi-site, confirmatory RCT that will be sufficiently powered. Estimates of potential recruitment/retention rates are based on cancer registry data and high recruitment rates at NCH for this population of caregivers. Estimated rates of recruitment are feasible considering there are approximately 80 HCT completed annually at NCH. Thus, there will be an ample number of eligible families to approach. We will compute effect sizes (e.g., standardized mean difference) using two-sided Type I error rates of α < .05 for the randomized group comparison to use for our future work assessing the efficacy of the intervention.

### Recruitment and randomization

Primary caregivers of children post-HCT will be approached in person after engraftment occurs and prior to the child’s discharge. If families express interest in participating, trained research staff will explain the purpose of the study and the study activities. Upon receiving confirmation from caregivers that they would like to participate, research staff will then guide them through the informed consent process (either written or electronic, whichever they prefer).

After informed consent is obtained, primary caregivers will be randomized to either the control (standard of care) group or the intervention (BMT4me© app) group. Randomization will be created in the online data collection tool REDCap^®^ [35] by the project’s statistician via a Randomization Module. The randomization sequence will be based on a design with blocks of four or six, chosen randomly within the sequence with equal probability. Randomly varying block sizes reduce the chance that research staff will guess the next group assignment, minimizing unconscious bias. The randomization sequence is protected and only the statistician will be able to edit it. However, study staff will have permissions to randomize and see the allocated group assignments when they log into REDCap^***®***^.

**Fig 1:**
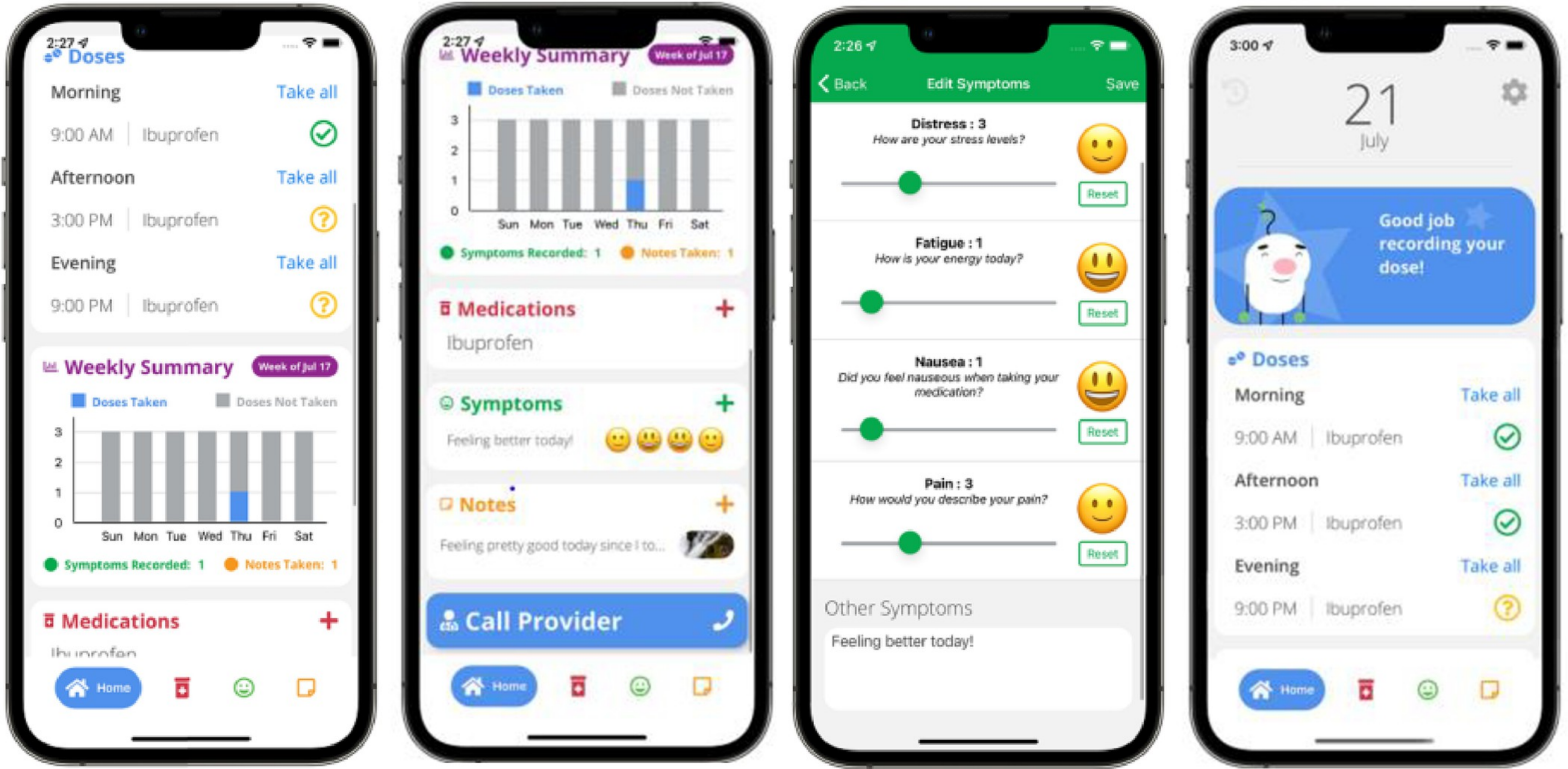
BMT4me app screenshots.

Following randomization, caregivers will be asked to complete baseline assessments **(Table 1**). All families will receive standard education at discharge with regards to medications and a medication list for their child. An electronic adherence monitoring device will be administered to all families at the beginning of the study. Caregivers of children prescribed immunosuppressant medications in a liquid form will receive a Medy remote patient management (MedyRPM) box [36]. MedyRPM will collect adherence data each time the box lid is opened and closed via a Bluetooth HUB and enabled monitor attached to the box [36]. Data will be transmitted via LTE connectivity cloud services and be available for viewing by study staff on a comprehensive patient management portal [36]. Caregivers of children who are receiving their immunosuppressant medication via pill or capsule will have the option of either using a MedyRPM box or medication event monitoring system*®* (MEMS) cap [37], whichever they prefer, for the duration of the study. MEMS*®* Caps have been used consistently to measure medication adherence [38, 39] by collecting data via a micro-electronic circuit which date/timestamps when the container is opened and closed. Study staff will download the data collected on the MEMS*®* cap weekly via the MEMS*®* adherence desktop software [37]. Additionally, caregivers assigned to the intervention group will have the BMT4me© app downloaded onto their personal device to log doses of medications. Brief follow-up assessments will occur weekly in person during participants’ clinical appointments or virtually, via email or telephone, whichever the family prefers, in both groups. Reasons for non-participation and dropout will be tracked. Each family will be given a $50 gift card at their time of enrollment, followed by another $25 at the end of the study, for a total of $75 in subject compensation. See **Fig 1** for a timeline of the study.

**Table 1:** Measure descriptions by visit.

Visit	Activity	Description
**Enrollment**	*Randomization *	Families will be randomized into one of two groups immediately following consent. If they are assigned to the intervention group, they will receive the mHealth app for medication tracking. If they are assigned to the control group, they will not receive the mHealth app.
	*Demographic Questionnaire *	Research staff ask participants to answer questions about age, race, ethnicity, religion, educational attainment, income (parents only), whom they live with, and their relationship to their child (parents only).
**Enrollment and Exit**	*Posttransplant Perception Survey*	A 4-item self-report clinical assessment tool asking participants to report on their views about their child’s health and perceptions post-transplant. Items are rated on a 5-point Likert scale. Administered at enrollment and exit of the study.
	*Barrier Scale *	A self-report clinical assessment tool with 14 commonly endorsed barriers. Domains include logistical issues (e.g., forgetting, inconvenience), ingestion difficulties (e.g., swallowing, taste), efficacy (e.g., feel I don’t need it), financial difficulties, regimen characteristics (e.g., too many medications, side effects), and patient-specific issues (e.g., refusal by child, embarrassment). Administered at enrollment and exit of the study.
**Weekly**	*Medication Adherence Measure (MAM) *	The MAM is semi-structured interview specific to pediatrics, conducted with the parent weekly, to obtain an individual score in each module. The score is represented in percentages of the number of required doses. A total summary score can be calculated across all medications, as well as separately.
	*Readmissions *	Readmission rates will be determined by the number of admissions requiring greater than a 24-hour stay within the first 100 days after discharge. Reason for readmission will be recorded and based on the EMR discharge diagnosis. Data will be collected weekly.
	*Graft vs*. *Host Disease (GVHD) *	GVHD will be assessed on the international standard acute GVHD grading and staging scale.55 Provider grading will be per organ system on a 1–4 scale, with an overall score collected weekly.
	*Immunosuppressant assay levels*	Immunosuppressant serum assays are collected weekly during the acute phase. A calculation of the degree of variation among the levels collected will be formulated to assess adherence to the prescribed immunosuppressant medication.
**3-Week Intervals**	*Pediatric Quality of Life Inventory (PedsQL) *	Parents will complete the Pediatric Quality of Life Inventory (PedsQL) every three weeks (at week 3, week 6, and week 9). The frequency of 23 problems in 4 domains (i.e., physical, emotional, social, school) are rated on a 3 or 5-point scale. Versions are based on child age: (a) 1–12, (b) 13–24 months old and (c) 2–4, (d) 5–7, (b) 8–12 and, (c) 13–18 years old.
**Monthly**	*Medy Remote Patient Management (RPM) medication box *	Medy RPM collects daily date and time-stamped data via an NFC reader. Each opening and closing is assumed to reflect an administered and consumed medication dose. This data will be collected monthly from participants as a direct measure of adherence.
	*MEMS Cap *	MEMS Caps collect daily date and time stamped data via a micro-electronic circuit that registers the opening and closing of the threaded pill bottle. Each opening and closing is assumed to reflect an administered and consumed medication dose. This data will be collected monthly from participants as a direct measure of adherence.
**Exit**	*Caregiver Satisfaction*	For the intervention group only, satisfaction will be assessed at the end of the study via semi-structured interviews and electronic surveys. Caregivers will be asked for feedback regarding participation in the intervention, benefit, burden, barriers, suggested modifications, and overall satisfaction. Suggested modifications to the app and advice to the healthcare team will also be solicited.

### Intervention

The BMT4me© app intervention was informed by Behavioral Economics (BE) Theory [40, 41] and the Pediatric Self-management Model [42]. Stakeholder feedback of wireframes was conducted through a mixed methods usability study and informed the initial development of the app. Then, the intervention was tested in a pilot study of primary caregivers of children discharged after HCT in a quasi-experimental pre-post design [43]. Caregiver feedback during final qualitative interviews were utilized to further modify the app, resulting in the current intervention (BMT4me 2.0©) employed within the scope of this RCT.

**Fig 2:**
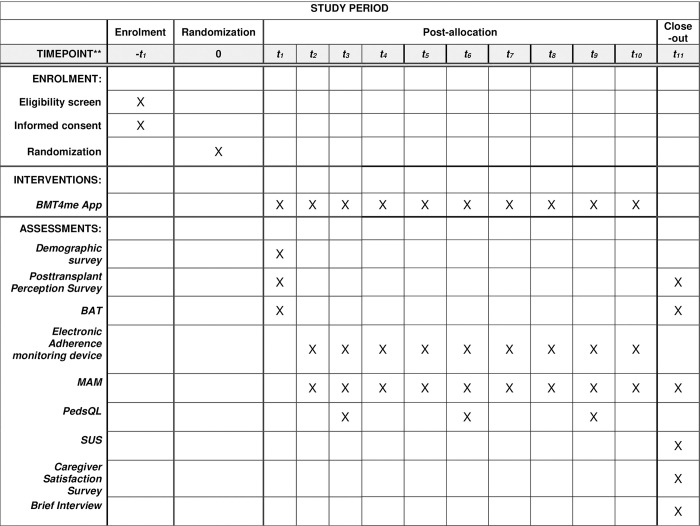
Schedule of enrollment, interventions, and assessments.

BMT4me 2.0© (**Fig 2**) was designed to aid in the management of medications, improve adherence monitoring, and track symptoms or medication side effects in real-time [43]. The app’s push notification feature reminds users of medication doses, utilizes ecological momentary assessment (EMA) [44] to record the time a medication was taken, and prompts users to provide reasons for missed doses. Users can add medications by entering them directly into the app or by capturing an image of the prescription with their phone camera with the image-to-text feature. Additionally, users can record symptoms and severity on a 1-to-10 sliding scale, with corresponding changing emojis. These visual aids make symptom entry more convenient and easily understandable for app users. Other features include a notes page for recording details of care, as well as the ability to upload photos and weekly summaries that can be converted to pdf format and shared with providers. The app is available for download on both iOS and Android devices in English language.

After randomization, caregivers randomized to the BMT4me*©* app will have the application installed on their personal cell phone device prior to discharge at no cost. Research staff will conduct a brief tutorial on functions and demonstrate use. These sessions will be audio-recorded (treatment fidelity). The caregiver will add immunosuppressants and the schedule for administration with oversight by the primary discharge nurse to ensure accuracy. Accuracy of medications within the app will be verified and recorded at each study visit, conducted during the child’s weekly clinic appointment (fidelity check). Caregivers will receive a reminder when their child’s immunosuppressant medication is due based on their chosen medication administration schedule.

### Data collection

Data will be collected at the following time points from both groups; discharge, weekly for the first 100 days (i.e., approximately 11 weeks) post-discharge, or until the participant’s child completes their taper from immunosuppressant medication, and monthly (for approximately 3 months). Specifically, the following data will be collected: baseline medical information (i.e., diagnosis, age at diagnosis, treatment history, medications), demographic characteristics, perceptions post-HCT, barriers to medication adherence, serum immunosuppression assays, caregiver reports of adherence to medications, and caregiver proxy report of child quality of life. Monthly electronic adherence monitoring devices and clinical outcomes data will also be collected in both groups. At the conclusion of the study, caregivers in the intervention group will complete the System Usability Scale. Caregivers in both groups will participate in a 15–30 minute semi-structured interview addressing: 1) experience with adherence post-transplant and participation in the trial (e.g., benefit, burden, barriers, satisfaction) and 2) caregivers in the app arm will be asked to share any suggestions to improve the BMT4me*©* app. Measures are described in **Table 1**.

### Outcomes

The primary expected outcome of this study is that caregivers will report above average acceptability (≥ 68%) of the BMT4me*©* app and those families randomized to receive the BMT4me*©* app will have higher medication adherence frequency than the standard of care group. The secondary expected outcome is that those children whose caregiver was randomized to receive the BMT4me*©* app will have less GVHD and fewer readmissions compared to the standard of care group. Descriptive statistics, correlations, and independent samples t-tests will examine the primary and secondary outcomes. The goal of this intervention is to increase adherence to immunosuppressant medication and improve key clinical outcomes such as graft vs. host disease and readmissions relative to standard care.

### Ethics

The Institutional Review Board (IRB) at NCH approved the project (#STUDY00002478) on March 22, 2022. Eligible participants include caregivers aged 18 or older. All eligible participants will provide written informed consent via pen and paper or electronically through an electronic signature module on REDCap [35] prior to study participation. This trial is registered in ClinicalTrials.gov (identifier NCT05515497). At the conclusion of the study, data will be made available and results from the trial will be submitted to ClinicalTrials.gov.

Due to the sensitive nature of the project, staff members have and will continue completing extensive training in research ethics both internally through NCH’s Learning Center modules and externally through the Collaborative Institutional Training Initiative (CITI) [45] online training portal. Moreover, staff will be closely supervised by the primary investigator (PI) who has extensive experience working with families and children undergoing HCT. The only authors of this manuscript who have access to information that could identify individual participants prior to, during, or after data collection are members of the research team who have received approval from the IRB at NCH.

## Statistical analyses

### Quantitative

During the passive use observation period, passive data modules will capture phone activity and caregivers’ application use (e.g., time/date, duration of use). Descriptive statistics will be used to analyze phone activity. Correlations will be used to investigate app use behavior over time. Acceptability will be assessed by averaging total scores from the system usability scale (SUS). Consistent with the literature [46–48], scores greater than 68% on the SUS will be considered acceptable [49]. The proportion of participants that enroll and complete the study will be examined to assess study feasibility.

The potential efficacy of the BMT4me© app will be examined using an independent samples t-test where the outcome is the proportion of adherence (i.e., the number of doses taken divided by the number of doses prescribed). If adherence is substantially non-normal, efficacy will be examined using an analogous logistic regression model. GVHD stages will be compared between the intervention and usual care groups using a chi-square analysis given the ordinal nature of this variable, whereas number of readmissions will be compared across the two groups using an independent samples t-test. If the number of readmissions is substantially non-normal, an analogous Poisson regression model will be used. Because the nature of the study is exploratory rather than confirmatory, the objective of the analysis is effect size estimation rather than formal hypothesis testing, and threats to power (e.g., participant attrition due to patient death, early taper) are not a primary concern. We will compute effect sizes (e.g., a standardized mean difference) for the randomized group comparison to inform our future work assessing the efficacy of the intervention on adherence to immunosuppression medication. Moreover, an exploratory aim will examine moderators by adding an interaction term and corresponding main effect for the moderator to the statistical model via the statistical software Mplus.

### Qualitative

Interviews will be audio-taped and transcribed verbatim then imported into NVivo [50]. The qualitative data analysis computer software within NVivo will then be utilized for content analysis using the constant comparison method, by at least two independent, trained, doctoral level coders. Study staff will read with immersion (i.e., repeatedly reading a subset of transcripts), cluster similar ideas to inform preliminary categories, and review and revise coding schemes. Study staff will then apply the codes to a second subset of transcripts, revise them as necessary, and repeat this process until saturation is reached [51–53]. Member checking will be completed with a subsample to obtain family input on thematic codes as a final validity check. Frequency counts of final themes will be obtained [52].

## Discussion

While long-term survival rates and better clinical outcomes have increased in pediatric hematological and oncological populations due to innovative therapies, poor adherence persists [54]. Reasons for non-adherence are complex, but may be due to levels of caregiver engagement, age of child, social factors, and cognitive factors [55]. Thus, with nearly 2,500 children receiving HCT annually [56]- and the increased societal burden (e.g., increases in costs of care and need for more clinicians to care for children due to the occurrence of adverse outcomes as they relate to non-adherence) associated with the higher number of transplants each year [57–59] there is a need for research examining potential targeted interventions to address non-adherence to medication regimens. The strengths of this RCT and digital health intervention include constructs from behavioral health and BE theory, which drive data collection and analysis [43], as well as the use of a mixed methods approach, which will allow for a comprehensive understanding of the acceptability and efficacy of the BMT4me*©* app intervention and the feasibility of enrolling 50 caregivers of children discharged following HCT. Additionally, the intervention has undergone numerous rounds of testing with families and stakeholders (i.e., HCT families, physicians, and nurses) whose unique and vital perspectives have informed its development to make it more desirable to HCT families. Finally, the universally accessible nature of the intervention means that families’ sociodemographic factors (e.g., distance from hospital/treatment site, access to reliable transportation and Wi-Fi, affordability of the intervention, etc.), which have been reported as barriers in the implementation of other interventions [60, 61], are not applicable within the context of this study.

This study design is not without potential limitations. First, participation may be burdensome; however, assessments are purposefully brief to reduce burden and caregivers will have the option of completing paper/pencil or electronic measures. Participants may also complete measures virtually via REDCap link or over the phone with study staff so their weekly clinic appointments are not impeded. Although some families may not have smartphones, we do not expect access to technology will be a significant exclusionary factor, as >90% of adults in the US own a mobile device [62]. If access is a problem, smartphones will be provided to families. Finally, the intervention is only available to English-speaking caregivers at this time, but opportunities to expand the intervention’s inclusivity (i.e., adding additional languages) are being pursued.

## Conclusion

In this protocol, we describe an RCT to evaluate the acceptability and efficacy of the newly developed BMT4me*©* app, as well as the feasibility of recruiting and retaining 50 caregivers of children who have been discharged during the acute phase post-HCT. Based on behavioral economics theories and adherence literature, we predict that this novel intervention will assist families in managing post-discharge medications, increase their adherence to medical regimens, and mitigate the onset of GVHD and subsequent readmissions. It is our understanding that this is the first prospective, longitudinal RCT examining the effects of the BMT4me*©* smartphone application intervention on adherence to immunosuppressant medications in the pediatric HCT population. Ensuing outcomes will address critical gaps in non-adherence knowledge and inform future studies of digital intervention outcomes in pediatric HCT.

## Supporting information

S1 File SPIRIT checklist. (DOCX)

S2 File Study protocol. (DOCX)
