# Supplementary material for: Improving medication adherence monitoring and clinical outcomes through mHealth: A randomized controlled trial protocol in pediatric stem cell transplant
Source: PLoS One. 2023 Aug 17;18(8):e0289987. doi: 10.1371/journal.pone.0289987 (PMC10434937; doi:10.1371/journal.pone.0289987)
Supplement: S1 File — (DOCX) [file pone.0289987.s001.docx]

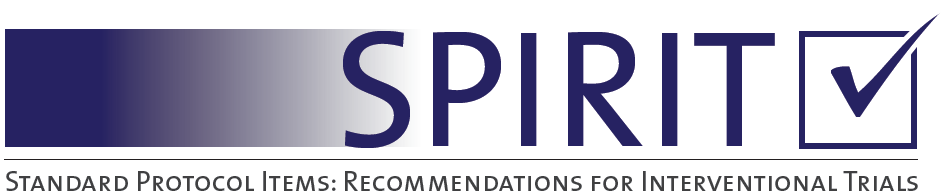


SPIRIT 2013 Checklist: Recommended items to address in a clinical trial protocol and related documents*

| Section/item | Item No. | Description |
| --- | --- | --- |
| **Administrative information** | | |
| Title | 1 | Improving Medication Adherence Monitoring and Clinical Outcomes through mHealth: A Randomized Controlled Trial Protocol in Pediatric Stem Cell Transplant |
| Trial registration | 2 | ClincialTrials.gov  NCT05515497 |
| Protocol version | 3 | Issue Date: 10 Feb 2022  Protocol Amendment Number: N/A  Author(s): *M.S.; J.R.*  Revision Chronology:   \| Original \|  \| \| --- \| --- \| \| 2022-Aug-16 \| Amendment 01: We updated our consent process to include the electronic signature (eConsent) module on REDCap. \| \| 2022-Aug-24 \| Amendment 02: We included three additional questionnaires (Posttransplant Perception Survey, Barrier Scale, Caregiver Satisfaction) to administer to families. \| \| 2022-Sep-6 \| Amendment 03: We updated eligibility criteria to include children ages 2-18 years of age. We also increased compensation for participation at enrolment from $25 to $50. \| \| 2022-Oct-6 \| Amendment 04: We updated eligibility criteria to include children ages 0-18 years of age. We also included information regarding REDCap text links and a new electronic adherence device (Medy Remote Patient Management medication box). \| \| 2023-Jan-16 \| Amendment 05: We updated eligibility criteria to include children and adolescents ages 0-21 years old and require patients be residing with a primary caregiver before enrolment onto the study. We also increased the sample size from 40 participants to 50. \| \| 2023-Mar-15 \| Amendment 06: We updated the end of study criteria to conclude study participation with a caregiver after their child has *completed* their wean/taper from immunosuppressant medication as opposed to concluding participation when their child begins their wean/taper. \| |
| Funding | 4 | The Oncology Nursing Foundation and National Institute of Health/Nursing (K99NR019115) supported the mHealth app prototype development. This study is supported by the National Institute of Health/Nursing (R00NR019115) |
| Roles and responsibilities | 5a | MS conceived of the study and CG, WL, AP, SL, JS, JR, and ES assisted with the study conceptualization. MS acquired funding for the project. MS, CG, WL, AP, SL, JS, JR, and ES contributed to the methodology. MS, AO, JER, AB and PG will help with project administration. MS and CG will supervise the project. MS, JER, and CS wrote the original draft, and CG, WL, AP, SL, JS, JR, and ES reviewed and edited the manuscript. |
|  | 5b | Dr. Skeens (Principal Investigator) is overseeing this trial at The Abigail Wexner Research Institute at Nationwide Children’s Hospital. 700 Children’s Dr. Columbus, OH 43205; 614-722-4502; micah.skeens@nationwidechildrens.org |
|  | 5c | The study sponsor and funders had no role in study design, collection, management, analysis, and interpretation of data; writing of the report; and the decision to submit the report for publication |
|  | 5d | **Principal Investigator**  Design and conduct of intervention  Preparation of protocols and revisions  Oversight of data management team  Communication with oncology team  Analysis of data  Publication of study reports    Research Assistants  Data collection, entry, and management  Analysis of data  Publication of study reports |
| **Introduction**  Background and rationale | 6a | Introduction (Pages 4-6). In the United States, poor adherence accounts for up to 70% of all medication-related hospital admissions, resulting in $100 billion in healthcare costs annually  Mechanisms. Adherence rates have been reported as low as 0% in pediatric patients. Reasons for non-adherence are multifactorial. The most important determinants of non-adherence are consistently documented as complexity and duration of treatment regimens, as well as forgetfulness. Thus, children undergoing difficult hematopoietic stem cell transplants (HCT) that require medication indefinitely are at high risk for medication non-adherence.  Existing Knowledge. Only 4 published studies exist regarding adherence in pediatric HCT. None address adherence to immunosuppressant medication, nor are they RCTs. Second, the complexity of most interventions for adherence is counter to the geographic, resource, and time constraints families of chronically ill children face. Adherence interventions based on conventional behavior theory have been cumbersome for families already stressed due to chronic illness. BE design is a significant paradigm shift to a simpler, less onerous approach that can engage those patients and families that would otherwise forego complicated adherence interventions. Although mHealth adherence apps are a widely available, simple, and innovative approach to addressing these problems, a third gap relates to poor usability. For example, a recent review of pediatric adherence apps found that none identified individual barriers to adherence, and nearly all were designed for adults. Thus, there is an urgent need to develop and evaluate innovative, accessible, and evidence-based approach to adherence among children receiving HCT to prevent morbidity and mortality from GVHD.  The impact of non-adherence on clinical outcomes is largely unknown in pediatric HCT. Poor adherence is generally associated with adverse outcomes, including complications, hospital admissions, and even death. The societal burden of cancer care and HCT is substantial and likely to increase based on the growing number of transplants each year. Clinicians and researchers have focused on GVHD prevention to minimize unnecessary treatment-related deaths. Acute GVHD develops in the first 100 days post-transplant. Children that develop acute GVHD have a 30% to 50% chance of survival. Morbidity and mortality due to GVHD can be decreased through prophylactic use of immunosuppressants. Although these medications are costly and produce unpleasant side effects, adherence is critical to decrease complications, reduce readmissions, and ultimately increase quality of life and survival.  Need for trial. Limited data exists regarding adherence in pediatric HCT. Additionally, broad gaps exist with regard to immunosuppressant medication adherence, nor does the HCT literature include adherence randomized control trials (RCT). Thus, this prospective longitudinal pilot RCT will evaluate the preliminary acceptability and efficacy of the mHealth app (BMT4me) and the feasibility of enrolling and retaining 50 caregivers of children outpatient during the acute phase post-HCT. |
|  |  |  |
|  | 6b | Participants in the control group will not receive the BMT4me app in order to test the efficacy of the intervention on adherence to immunosuppression medication post-discharge. |
| Objectives | 7 | - 1. Research Hypothesis (see page 5-6 of the manuscript)   Caregivers enrolled in this study are hypothesized to:   - Report above average acceptability (> 68%) of the newly developed mHealth app, and > 75% of participants will enroll and complete all study-related assessments, - Have higher adherence frequency, if randomized to the mHealth app, than the standard of care group, and - Have less GVHD and fewer readmissions than the standard of care group.   1. Study Objectives      1. Primary Objective   The primary objective is to evaluate the acceptability of the newly developed mHealth app (BMT4me) and the feasibility of enrolling and retaining 50 caregivers of children in the acute phase post-HCT in a small-scale RCT.   - 1. Secondary Objectives      1. Key Secondary Objectives   Evaluate the potential efficacy of a mHealth app on adherence to immunosuppressants in children who have been discharged home during the acute phase post-HCT.   - - 1. Other Secondary Objectives   To explore, in caregivers of paediatric-HCT recipients, the newly developed mHealth app in regard to:   - Reports of acceptability (> 68%), and participant retention (i.e., completion of all study-related assessments), - Determine if randomization to the mHealth app is associated with higher adherence frequency than those participants randomized to the standard of care group, and - Assess if randomization to the mHealth app is associated with lower rates of GVHD and readmission(s) in children than the group receiving standard of care. |
| Trial design | 8 | (see page 8 of the manuscript) The mobile health (BMT4me) intervention is designed as a randomized and controlled trail. Randomization will be created in the online data collection tool REDCap^®^. by the project’s statistician via a Randomization Module. The randomization sequence will be based on a design with blocks of four or six, chosen randomly within the sequence with equal probability. Randomly varying block sizes reduce the chance that research staff will guess the next group assignment, minimizing unconscious bias. The randomization sequence is protected and only the statistician will be able to edit it. However, study staff will have permissions to randomize and see the allocated group assignments when they log into REDCap**^®^**. |
| Methods: Participants, interventions, and outcomes | | |
| Study setting | 9 | (see page 7 of the manuscript) Participants will include 50 caregivers of children who received HCT. Children meeting eligibility criteria (described in section 10) will be identified through weekly inpatient HCT unit rosters. Caregivers will be recruited prior to their child’s discharge from the inpatient HCT floor at Nationwide Children’s Hospital, a large pediatric academic medical center in the Midwest. |
| Eligibility criteria | 10 | (see page 7 of the manuscript) Caregivers must provide written or electronically signed informed consent prior to any study visit procedures.  10.1 Inclusion Criteria  Children of caregivers must be:   1. 0 to 21 years of age 2. Receiving immunosuppression for an allogenic transplant 3. Discharged prior to Day 100 or immunosuppression taper 4. Residing with the primary caregiver that enrolls on study   Primary caregivers must be:   1. English-speaking 2. Have an iOS or Android capable cellular device   10.2 Exclusion Criteria   1. Caregivers of children with a documented developmental delay will be excluded. 2. Adults unable to consent will be excluded. |
| Interventions | 11a | (see pages 9-11 of the manuscript) Eligible caregivers will be randomized to either the intervention (mHealth app) or standard of care and will complete baseline assessments. The randomization sequence will be based on a design with blocks of four or six, chosen randomly within the sequence with equal probability. A pseudorandom number generator in REDCap will be used, and the randomization sequence will be maintained by the statistician. Randomly varying block size reduces the change that research staff will guess the next group assignment, minimizing unconscious bias.  Once randomized, those caregivers randomized to the mHealth app will have the application installed on their personal cell phone device at no cost prior to discharge. Research staff will conduct a brief tutorial on functions and demonstrate use. These sessions will be audio-recorded (treatment fidelity). The caregiver will add immunosuppressants and the schedule for administration with oversight by the primary discharge nurse to ensure accuracy. Accuracy of medications within the app will be verified and recorded at each visit (fidelity check). Caregivers will receive a reminder when the immunosuppressant medication is due. The caregiver will then log within in the app whether the medication was given, as well as any symptoms that occurred around the time of the medication’s administration. If the medication is not given, the caregiver will document an explanation as to why. |
|  | 11b | NA |
|  | 11c | (see pages 7-10 of the manuscript) Participants will receive an electronic adherence monitoring device and/or the newly developed mHealth (BMT4me) app- depending on the group they are randomized to- at discharge (i.e., the conclusion of their initial study visit) in the following manner:  All families will receive standard education at discharge with regards to medications and a medication list for their child. An electronic adherence monitoring device will be administered to all families at the beginning of the study. Caregivers of children prescribed immunosuppressant medications in a liquid form will receive a Medy remote patient management (MedyRPM) box. MedyRPM will collect adherence data each time the box lid is opened and closed via a Bluetooth HUB and enabled monitor attached to the box.[36] Data will be transmitted via LTE connectivity cloud services and be available for viewing by study staff on a comprehensive patient management portal. Caregivers of children who are receiving their immunosuppressant medication via pill or capsule will have the option of either using a MedyRPM box or medication event monitoring system® (MEMS) cap, whichever they prefer, for the duration of the study. MEMS® Caps have been used consistently to measure medication adherence[38,39] by collecting data via a micro-electronic circuit which date/timestamps when the container is opened and closed. Study staff will download the data collected on the MEMS® cap weekly via the MEMS® adherence desktop software.  Additionally, caregivers assigned to the intervention group will have the BMT4me© app downloaded onto their personal device to log doses of medications.  Participant data collected from the MEMS cap or MedyRPM box will be collected monthly from both groups. Accuracy of medications within the BMT4me app for the intervention group will be verified and recorded at each study visit.  **Adherence assessments**  Numerous methods will be utilized to assess medication adherence including an electronic adherence monitoring device (MEMS cap or MedyRPM box) and the newly developed mHealth BMT4me app. All of the electronic adherence monitoring devices have been independently tested for accuracy. The BMT4me app was designed by the AWRI RISI developers at Nationwide Children’s Hospital and has been through multiple phases of stakeholder testing.  Electronic data collected by either the MEMS® Cap or MedyRPM box and BMT4me app will be downloaded using cloud or computer-based software onto secure hospital servers, which are only accessible by research staff. |
|  | 11d | Standard discharge education with regards to medications will be provided to participants in both the control and intervention arm. No intervention is prohibited. |
| Outcomes | 12 | (see Table 1)   1. Primary outcome measures:  - Electronic Adherence Monitoring Device [Time Frame: Baseline (Day 0) to Day 100]   - Medy Remote Patient Management (RPM) medication box by Vaica or Medication Event Monitoring System (MEMS) Cap. - Medication Adherence Measure (MAM) [Time Frame: Baseline to Day 100] - System Usability Scale (SUS) [Time Frame: Exit (Day 100)] - Caregiver Satisfaction [Time Frame: Exit (Day 100)]  1. Secondary outcome measures:  - Demographic Data Form [Time Frame: Baseline (Day 0)] - Medication Possession Ratio (MPR) [Time Frame: Monthly until Day 100] - Medication Level Variability Index (MLVI) [Time Frame: Weekly until Day 100] - Graft vs. Host Disease (GVHD) [Time Frame: Weekly until Day 100] - Readmissions [Time Frame: Weekly until Day 100] - Posttransplant Perception Survey [Time Frame: Baseline (Day 0) and Exit (Day 100)] - Barrier Assessment Tool (BAT)- Caregiver [Time Frame: Baseline (Day 0) and Exit (Day 100)] - Pediatric Quality of Life Inventory (PedsQL) version 4.0. [Time Frame: Week 3 (Day 30), Week 6 (Day 60) and Week 9 (Day 90)] |
| Participant timeline  (Fig.1) | 13 | 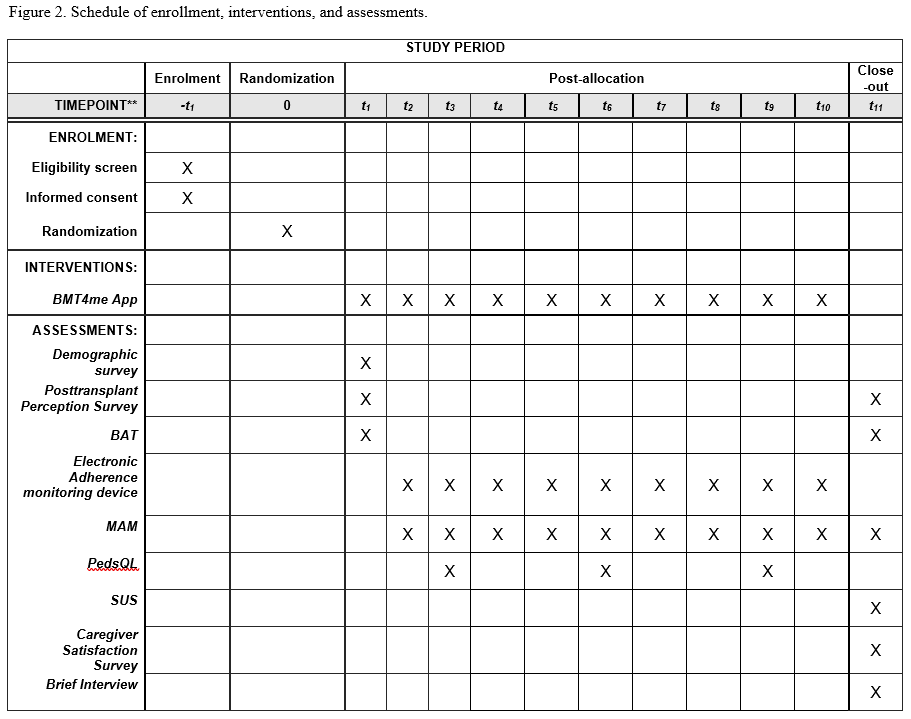  The primary variable of interest is adherence to immunosuppressant medication post-HCT. Secondary outcomes include participant recurrence of GVHD and readmission rates. Data will be collected via self-report of caregivers. Additional relevant clinical information will be reviewed and extracted via electronic medical records. Finally, data will be collected via the BMT4me app and either a MEMS cap or MedyRPM box. All digital data collected via MEMS caps/MedyRPM box and/or the BMT4me app regarding participant usage will **not** be shared with providers while the study is active.  **13.1.1 Questionnaires**   - Demographic Data Form- Caregiver [Time Frame: Baseline]   - The caregiver will report on basic background characteristics including parent and child age, sex, race, ethnicity, education level, and family income. - Medication Adherence Measure (MAM)- [Time Frame: Weekly]   - The MAM is semi-structured interview specific to pediatrics, conducted with the parent, to obtain an individual score in each module. The score is represented in percentages of the number of required doses. A total summary score can be calculated across all medications, as well as separately. This allows for quantification of the degree of adherence on a continuum. MAM has demonstrated adequate convergent validity with MEMs caps (r =−.40, p < .05). - System Usability Scale (SUS)- [Time Frame: End of Study]   - The SUS is a 10-item questionnaire routinely used to evaluate the functionality and acceptability of mHealth apps. Items are rated on a 5 point scale and scores range from 0 to 100. Reliability (0.91) and validity (.81 correlation with 7- point scale of “user friendliness”) have been well established. A score of > 68% is considered above average.   **13.1.2 Additional Interviews/Measures of Adherence**   - Medication Possession Ration (MPR)- [Time Frame: Monthly]   - MPR is a standard adherence measure that uses pharmacy refill records to calculate adherence using the sum of the days’ supply obtained between the first pharmacy fill and the last fill divided by the total number of days. Higher scores indicate better adherence. The number of refills increases accuracy of the adherence estimate. - Medy Remote Patient Management (RPM) medication box- [Time Grame: Daily from Baseline to End of Study]   MedyRPM collects daily data via an NFC reader. Each opening and closing are assumed to reflect an administered and consumed medication dose. This data will be collected monthly from participants as a direct measure of adherence. Electronic monitors contain micro-electronic circuits that date and timestamp each time the container is opened to remove a dose of the medication. Data from the electronic monitors will be downloaded using cloud-based software at each study visit. An adherence percentage will be calculated by dividing the number of doses taken by the number of doses prescribed for each day. All of the electronic monitors have been independently tested for accuracy.   - Electronic Medication Event Monitoring System (MEMS) Cap- [Time Frame: Daily from Baseline to End of Study]   - MEMS Caps collect daily data via a micro-electronic circuit that registers the opening and closing of the threaded pill bottle. Each opening and closing is assumed to reflect an administered and consumed medication dose. This data will be collected monthly from participants as a direct measure of adherence. Electronic monitors contain micro-electronic circuits that date and timestamp each time the container is opened to remove a dose of medication. Data from the electronic monitors will be downloaded using cloud or computer-based software, at each study visit [Wians][Andrade, et.al.]. An adherence percentage was calculated by dividing the number of doses taken by the number of doses prescribed for each day. All of the electronic monitors have been independently tested for accuracy [Hommel et.al]. - Medication Level Variability Index (MLVI)- [Time Frame: Weekly]   - The MLVI is the calculation of the standard deviation of serum assays of immunosuppressants that has shown to correlate with adherence and clinical outcomes in the solid organ transplant population.54 Immunosuppressant serum assays are collected weekly during the acute phase. A calculation of the degree of variation among levels will be formulated. - Graft vs. Host Disease (GVHD)- [Time Frame: Weekly]   - GVHD will be assessed on the international standard acute GVHD grading and staging scale.55 Provider grading will be per organ system on a 1-4 scale, with an overall score given weekly. - Readmissions- [Time Frame: Weekly] - Readmission rates will be determined by the number of admissions requiring greater than a 24-hour stay within the first 100 days after discharge. Reason for readmission will be recorded and based on the EMR discharge diagnosis. In addition, a sub-analysis of readmissions within the first 30 days after initial discharge post-HCT will be completed. - Caregiver Satisfaction- [Time Frame: End of Study] - Satisfaction will be assessed via semi-structured interviews and electronic surveys with caregivers. Caregivers will be asked for feedback regarding participation in the intervention, benefit, burden, barriers, suggested modifications, and overall satisfaction. Suggested modifications to the app and advice to the healthcare team will also be solicited. |
|  |  |  |
| Sample size | 14 | (see page 7 of the manuscript) Given this is a pilot RCT, the overall goal is to examine preliminary efficacy and establish reliable effect sizes to inform a larger multi-site RCT that will be sufficiently powered. Thus, the sample of 50 (25 intervention, 25 standard of care only) caregivers. Estimates of potential recruitment/retention rates are based on cancer registry data and strong documented recruitment experience at NCH. With approximately 80 transplants annually at NCH, we will have ample eligible families to approach. The goal is to enroll at least 50 families, with approximately 20% attrition prior to completion of all measures for a total of 50 (~80% of enrolled dyads). We anticipate 85% mothers as primary caregivers, and children will be equally distributed by sex.  Potential efficacy of the intervention will be examined in the R00 phase using an independent samples t-test where the primary outcome is the proportion of adherence. If adherence is substantially non-normal, efficacy will be examined using an analogous logistic regression model. Because the nature of the study is exploratory rather than confirmatory, the objective of the analysis is effect size estimation rather than formal hypothesis testing, and threats to power (e.g., participant attrition due to patient death, early taper) are not a primary concern. We will compute effect sizes (e.g., a standardized mean difference) for the randomized group comparison to use for our future work assessing the efficacy of the intervention. |
| Recruitment | 15 | (see page 9 of the manuscript) Study staff will enroll at least 50 families, with approximately 20% attrition prior to completion of all measures for a total of 40 (~80% of enrolled dyads). We anticipate 85% mothers as primary caregivers, and children will be equally distributed by sex. Enrollment is expected to last three years.  Research staff will screen participants through EPIC, the hospital’s medical database. Study staff will continuously work on screening for eligible participants, conducting, and completing the study until the target population is achieved. Due to exclusion criteria, the cohort enrolled might not reflect all participants in our tracking file. Enrollment will extend over three years, or until our desired sample size is achieved. A $50 gift card will be provided to all families at enrolment and another $25 at the end of the study.  To ensure the privacy of patients while collecting PHI for eligibility purposes, a HIPAA Authorization form was obtained through the Institutional Review Board prior to the onset of the study. |
| **Methods: Assignment of interventions (for controlled trials)** | | |
| Allocation: |  |  |
| Sequence generation | 16a | (see pages 6, 8 of the manuscript) Participants will be randomly assigned to either control or experimental group with a 1:1 allocation by computer generated randomization stratified by age and gender with blocks of 4 and 6 chosen randomly (1:1) to either receive 1) standard discharge care and the mHealth “BMT4me” app intervention or 2) standard discharge care. |
| Allocation concealment mechanism | 16b | (see page 8 of the manuscript) Participants will be randomized using the randomization module in REDCap, a data management software. The randomization sequence will be maintained by the statistician. Allocation concealment will be ensured via randomly varying block sizes, which reduce the chance that research staff will predict the next group assignment prior to participant recruitment. |
| Implementation | 16c | All patients who consent to participating in the study will be randomized. Randomizations will be stratified by age and gender and completed in the REDCap randomization modules with blocks of four to six, chosen randomly within the sequence with equal probability. The randomization sequence will be maintained by The Center for Biobehavioral Health at NCH. Primary investigators and study staff will have no influence on group randomization. |
| Blinding (masking) | 17a | Study staff will be able to view the randomization sequences and group assignments in the REDCap online randomization module, where assignments will occur. Only the biostatistician will be able to edit the randomization module. Due to the project’s study design, study staff and participants cannot be blinded to group assignments. |
| Emergency Unblinding | 17b | Due to the nature of the intervention, there is no reason for a participant to need a new group assignment. If a participant wishes to be taken out of the intervention group, they may withdraw from the study. However, they would not be assigned to the control group as a result. |
| **Methods: Data collection, management, and analysis** | | |
| Data collection methods | 18a | (see pages 6-12 in the manuscript) Primary caregivers in both groups who consent to participate will complete baseline measures prior to discharge from the hospital. Families will be randomized (as described above) to receive the mHealth app or standard of care. Caregivers randomized to the mHealth app will have the application installed on their personal cell phone device at no cost. Research staff will conduct a brief tutorial on functions and demonstrate use. These sessions will be audio-recorded (treatment fidelity). The caregiver will add immunosuppressants and the schedule for administration with oversight by the primary discharge nurse to ensure accuracy. Accuracy of medications within the app will be verified and recorded at each weekly follow-up visit (fidelity check). The following data will be recorded on all patients; baseline medical information (i.e., diagnosis, age at diagnosis, treatment history, medications), demographic characteristics, perceptions post-HCT, barriers to medication adherence, serum immunosuppression assays, and caregiver reports of adherence to medications. Monthly MEMS Cap or MedyRPM box monitoring and clinical outcomes data will also be collected in both groups. Families will complete the study when they reach Day 100 or complete taper from immunosuppression, whichever is first. At the conclusion of the study, caregivers in the mHealth app group will complete the System Usability Scale. Caregivers in both arms will participate in a 15–30 minute semi-structured interview addressing: 1) experience with adherence post-transplant and participating in the trial (e.g., benefit, burden, barriers, satisfaction) and 2) Caregivers in the intervention group will be asked to share any suggested changes to the application. A $50 gift card will be provided to all families at enrollment and another $25 at the end of the study.  Rigor and reproducibility will be ensured through the randomized controlled design; a highly manualized approach to procedures for recruitment and data collection; multiple methods (qualitative and quantitative); standardized measures with strong psychometric properties; centralized/electronic data entry/management, and rigorous statistical tests of proposed hypotheses. M.S. will monitor the study to ensure intervention fidelity and adherence to regulatory and data management requirements. Interventionists will be trained, and the sessions will be audiotaped and double-coded by M.S. and C.G. to ensure fidelity. The team will discuss recruitment and protocol activities, including intervention fidelity, during regular lab meetings. |
| Retention | 18b | (see page 7 in the manuscript) Estimates of potential recruitment/retention rates are based on cancer registry data and strong documented recruitment experience at NCH. With approximately 80 transplants annually at NCH, we will have ample eligible families to approach. The goal is to enroll at least 50 families over 2.5 years. We anticipate 85% mothers as primary caregivers, and children will be equally distributed by sex.  Once a caregiver has consented to be in the study, study staff will prioritize following-up with the family for the duration of the study period, in order to retain as many participants as possible. Follow-ups will occur weekly in both groups. Monthly assessments on medication possession ratios and clinical outcomes data will also be collected. If measures are not being completed electronically, study staff will approach participants with weekly measures in clinic, as an alternative. If it is an inconvenient time for the family, study staff will coordinate a better time with the family to return. These follow-up visits will occur electronically or in-person for the duration of the study or until a family decides to withdraw. Participants will be considered “lost to follow-up” if study staff are unable to contact the participant after 3 consecutive phone calls, email, and/or in-person clinic attempts. Finally, participants can withdraw from the study at any time, without explanation. |
| Data management | 19 | (see pages 12, 13 of the manuscript)  **19.1 Quantitative Data**  *19.1.1 Data Forms and Data Entry*  All data will be collected electronically via an online survey tool, REDCap, on a study iPad. For those families who do not wish to complete their surveys electronically, measures will be administered via pen and paper. Data collected on pen and paper will later be manually entered into REDCap by a trained study staff member.  *19.1.2 Passive Use Observation Data*  During the passive use observation period, passive data modules will capture phone activity and caregivers’ application use (e.g., time/date, duration of use).  **19.2 Qualitative Data**  Semi-structured qualitative interviews will be conducted by trained research staff at the conclusion of the study with participants in both groups. These interviews will be audio-taped and subsequently transcribed verbatim for content analysis by at least two independent, trained, doctoral level coders.  **19.3 Data storage**  All data will be identified by subject numbers and forms that must contain identifying information (e.g., consent forms, recruitment records) will be stored separately from information identified by ID number. All paper data will be stored in secured, locked file space in the Center for Biobehavioral Health. Tracking sheets that link study ID numbers and identifiers will be stored on a secure server and only be accessible to the PI and staff involved in recruitment or specific data management tasks. Data collected online will be housed by a secure firewall on the NCH research server and stored on secure NCH computers. Only study personnel will be able to access this information. All study materials will be retained for at least 3 years, or after the formal conclusion of the study. Following the discontinuation of the project, all paper materials will be sent to NCH’s fireproofing, and held for a minimum of 6 years.  **19.4 Data Privacy**  *19.4.1 Participants*  Study participants will be informed through the consent/assent process that their data will be kept private either through subject numbers or by redaction of PHI mentioned in recorded interviews. Exceptions to child safety are disclosed at that time.  *19.4.2*  M.S. will monitor the study to ensure intervention and adherence to regulatory and data management requirements. The team will discuss recruitment and protocol activities, including intervention fidelity, during weekly team meetings. |
| Statistical methods | 20a | (see pages 16 & 17 of the manuscript)  **20a.1 Qualitative Analyses**  Interviews will be audio-taped then imported and transcribed verbatim in NVivo^1^ (version 12) for content analysis using the constant comparison method, by at least 2 independent, trained, doctoral level coders. In brief, we will begin with immersion (i.e., repeatedly reading a subset of transcripts), cluster similar ideas to inform preliminary categories, review and revise coding schemes, apply the coding scheme to a second subset of transcripts, revise themes, and repeat this process until reaching saturation and consensus. Member checking will be completed with a subsample to obtain family input on thematic codes as a final validity check, and frequency counts of final themes will be obtained.  **20a.2 Quantitative Analyses**  During the passive use observation period, passive data modules will capture phone activity and caregivers’ application use (e.g., time/date, duration of use). Descriptive statistics will be used to analyze phone activity. Correlation analysis will be used to investigate use behavior over time. Acceptability will be assessed by averaging total scores from the System usability scale. Consistent with the literature,^2^ scores > 68% on the SUS will be considered acceptable.  **20a.3 Power**  Given this is a pilot RCT, the overall goal is to examine preliminary efficacy and establish reliable effect sizes to inform a larger multi-site RCT that will be sufficiently powered. Thus, the sample of 50 (25 intervention, 25 standard of care only) caregivers. The potential efficacy of the intervention will be examined in the R00 phase using an independent samples t-test where the primary outcome is the proportion of adherence. If adherence is substantially non-normal, efficacy will be examined using an analogous logistic regression model. Because the nature of the study is exploratory rather than confirmatory, the objective of the analysis is effect size estimation rather than formal hypothesis testing, and threats to power (e.g., participant attrition due to patient death, early taper) are not a primary concern. We will compute effect sizes (e.g., a standardized mean difference) for the randomized group comparison to use for our future work assessing the efficacy of the intervention. |
|  | 20b | NA |
|  | 20c | In the very unlikely event a participant becomes overly distressed from the questions, the examiner will discontinue the protocol and refer the family to psychosocial services if desired. Reasons for non-participation and dropout will be tracked for the CONSORT diagram. Additionally, should participants withdraw after randomization, sensitivity analyses will be conducted to assess potential differences in focal analyses based on whether or not participants withdrew. Missing data will be assessed on a case-by-case basis using current missing data techniques (e.g., multiple imputation, full information maximum likelihood, prorated scoring, etc.). |
| **Methods: Monitoring** | | |
| Data Monitoring | 21a | A system for monitoring recruitment, data collection, and general conduct of the study will include detailed training, weekly lab meetings, and secured collection and storage of data will begin with the start of the project. In addition, all data will be checked in real time to ensure completeness, and if items are not completed, the reason will be documented. Any paper copies will be marked with ID numbers and stored in a secure location. All data will be entered into a database by a trained research assistant and verified by the PI. All data files will be maintained in REDCap (Research Electronic Data Capture), a central electronic database system securely stored at the Research Institute at NCH. The master file will be maintained in the PI’s password-protected computer. All Adverse events will be reported to the IRB when they occur to determine if new procedures need to be implemented. |
| Interim Analysis | 21b | We will not conduct planned interim analyses. |
| Harms | 22 | Per NCH IRB, this protocol defines an adverse event (AE) as (a) any unfavorable or unintended event causing any negative physical or emotional outcome that was not present at the time of enrollment, (b) an event that may occur even in the absence of any error or protocol deviation, and (c) does not necessarily have to be caused by any identifiable aspect of the research. A serious adverse event (SAE) is defined as any AE that results in hospitalization, permanent disability, or death.  Within the scope of this study, we believe that there is very little chance that any AEs or SAEs will occur as a result of participants being enrolled.  However, it is possible some participants may feel upset when answering questions about their child’s diagnosis or medical treatment; but it is more likely that they find the questions or feedback process to be boring. If any questions are found to be upsetting or participants do not want to complete a question, they will not be forced to, and a study team member will be available to discuss it with the participant further, if desired.  Finally, although every precaution will be taken, there is a small chance of loss of confidentiality of your study information |
| Auditing | 23 | Some data will be collected using paper documents, then manually entered in an electronic database using REDCap. These data points will be entered twice, each time by a different staff member. A third staff member will then conduct a data comparison check, to ensure all data was accurately entered electronically. Study staff will also regularly review and address any protocol deviations, if needed.  Additionally, interviews will be audio-taped and transcribed verbatim for content analysis using the constant comparison method, by at least two independent, trained, doctoral level coders. Member checking will be completed with a subsample to obtain family input on thematic codes as a final validity check, and frequency counts of final themes will be obtained.  Moreover, the PI will be responsible for continuously conducting data management assessments with the purpose of identifying and reporting an deviations, as is required by the IRB. Any independent monitoring that may become necessary throughout the life of the study will be accommodated by the PI throughout the direct sharing of all trial related data, documents, and reports. |
| Ethics and dissemination | | |
| Research ethics approval | 24 | All study materials- including study protocol, consent form(s), recruitment materials, and participant materials- will be submitted to the IRB for review and approval. Approval of all study materials will be obtained prior to participant consent and enrollment. Amendments to the study protocol will be submitted to the IRB for review and approval before implementation to the study occurs. Authorized entities of the study will be able to review all materials and records collected in the study and held by the primary investigator.  The study team will report any adverse events (AEs) to the NCH IRB annually. Unexpected serious adverse events (SAEs) related to the study intervention will be reported to the NCH IRB within 5 business days of discovery, per NCH HRP-503 (updated 12/10/18). Any additional SAEs will be reported to the NCH IRB annually and to NCI per their guidance. |
| Protocol amendments | 25 | NCH IRB defines a protocol deviation as “any noncompliance with the clinical trial protocol, International Council on Harmonization Good Clinical Practice (ICH GCP), or Manual of Procedures (MOP) requirements”. Study staff will regularly review and address any protocol deviations, if needed. Corrections will be implemented when necessary, depending on the nature and severity of the deviation.  Noncompliance- a failure to follow the regulations, requirements, or determinations of the IRB- may occur by a participant, the study investigator, or study staff. In preparation for the unlikely event that noncompliance will occur, corrective action plans will be developed and, when necessary, employed. |
| Consent or assent | 26a | (see page 8 of the manuscript) Consent documents (obtained with pen and paper or electronically on an iPad via REDCap electronic signature module) describing the study intervention, procedures, and possible risks will be administered prior to a participant’s enrollment on the study or randomization to the study intervention.  Caregivers will provide informed consent, during which a study team member will describe the scope of the study, their rights as participants, and go over any questions prior to beginning data collection. All consented caregivers will be given a blank paper or electronic copy of the signed consent form for their records. Participants will be reminded that their participation is voluntary throughout the duration of their time in the project.  All consent will take place in-person, in the inpatient bone marrow transplant (BMT) clinic. Participants will be given a copy of the consent form to follow along with as a study member reviews it. Then, study staff will collect the caregiver’s written or electronic signature; assent will not be required within this study, as no minors will be enrolled. All consent forms will be collected following NCH IRB guidelines and policies outlined in SOP: Informed Consent Process for Research (HRP-090) to ensure participants understand the voluntary nature of their participation and do not feel coerced into participation. |
|  | 26b | NA |
| Confidentiality | 27 | All study visits will be conducted in a private setting. Any data collected during study visits that could contain identifiable information will be stored in locked cabinets and/or secure hospital servers which are only accessible by research staff, authorized representatives of the IRB, or the study sponsor. Data will be stored until the research is completed and may be stored longer, according to NCH regulations. The primary investigator will be responsible for receipt or transmission of the data. No data collected at NCH will be transported or shared with unauthorized third-party personnel without prior approval from the funding agency.  A system for upholding the confidentiality of all data collected will include detailed trainings, weekly lab meetings, and secured collection and storage of data. All data will be checked in real time and any PHI included in the data will not have the general participant ID marked on them. In addition, data will be stored in a secure location, separate from the list of study IDs and participant data so that PHI cannot be linked. Other types of data, such as qualitative interviews, will be transcribed verbatim and any identifying information included in them will be removed from the transcripts. Audio files will be deleted after data collection is completed.  Data collected on paper copies will be entered into a database by a trained research assistant and verified by the PI. All data files will be maintained in REDCap (Research Electronic Data Capture), a central electronic database system securely stored at the Research Institute at NCH. The master file will be maintained in the PI’s password-protected computer. |
| Declaration of interests | 28 | The study’s principal investigator and other study staff have no financial or competing interests to disclose in relation to this project. |
| Access to data | 29 | **29.1 Access to Data**  Per NIH policy, all study results and accomplishments that it funds should be made available on the NIH grants public website (<https://grants.nih.gov/policy/sharing.htm>). If any data are shared, the PI will implement safeguards for the protection of privacy, confidentiality, and security for all dissemination and reproducibility purposes. All identifiables will be thoroughly removed from data and any shared data will not be traceable to study participants. The PI will follow NCH’s guidelines for data storage (e.g., saved on a secure server until data collection is complete, then moved to fireproofing for 5 years following the close of the study). Additionally, publication and data sharing of this study will be compliant with the following NIH policies:  **29.2 NIH Policies on data sharing and publication.**  *29.2.1 NIH Public Access Policy.* This study will comply with the National Institutes of Health (NIH) Public Access Policy, which requires that “research papers describing research funded by the National Institutes of Health be available to the public free through PubMed Central within 12 months of publication” (<https://publicaccess.nih.gov/policy.htm>).  *29.2.2. NIH Data Sharing, Dissemination, and Submissions Policies.* This study will also follow the NIH’s Data Sharing Policy and Policy on the Dissemination of NIH-funded Clinical Trial Information and the Clinical Trials Registration and Results Information Submission rule. Per these policies, the study’s PI will register this trial and submit study results to ClinicalTrials.gov. The PI and study team members will also attempt to publish all study findings in peer-reviewed academic journals. |
| Ancillary and post-trial care | 30 | N/A |
| Dissemination policy | 31a | Study results will be submitted to the Clinical Trials website (ClinicalTrials.gov) no later than one year post-data collection. Submission information will include participant flow information, demographic and descriptive information of enrolled participants upon enrolment, results of primary and secondary outcomes, and any AEs or SAEs that occurred. Dissemination of study findings will also occur via conference presentations and published manuscripts in academic journals of the Primary Investigator’s choosing. |
|  | 31b | Publications will be developed by study staff, with no use of professional writers. The PI will suggest topics for presentation and publication. The PI and research staff will seek opportunities to present/publish research findings 2-3 times per year; conferences will include International Society of Pediatric Oncology (SIOP); Association of Pediatric Hematology/Oncology Nurses (APHON) and American Society for Transplant and Cellular Therapy (ASTCT). In addition to the protocol paper, we intend to publish a manuscript on the technical development of the app in Year 2, as well as preliminary findings from the previous pilot study. Findings from this study will be summarized at the end of Year 3 and include papers on adherence monitoring, adherence in pediatric hematopoietic stem cell transplant patients and outcomes of the novel mHealth intervention on adherence monitoring among pediatric HCT. An R01 will be submitted at the beginning of Year 4 to further test the intervention in a larger multi-site RCT. |
|  | 31c | Dr. Skeens, the project’s Principal Investigator, has registered this clinical trial in ClinicalTrials.gov. All descriptive, recruitment, location, contact, and administrative information can be located by accessing the Clinical Trials website and searching for this project’s trial registration number (NCT05515497). |
| Appendices |  |  |
| Informed consent materials | 32 | See Appendix A |
| Biological specimens | 33 | N/A |

*It is strongly recommended that this checklist be read in conjunction with the SPIRIT 2013 Explanation & Elaboration for important clarification on the items. Amendments to the protocol should be tracked and dated. The SPIRIT checklist is copyrighted by the SPIRIT Group under the Creative Commons “[Attribution-NonCommercial-NoDerivs 3.0 Unported](http://www.creativecommons.org/licenses/by-nc-nd/3.0/)” license.

References

1. International Q. 2021.

2. Lewis JR, Sauro J. The Factor Structure of the System Usability Scale. 2009; Berlin, Heidelberg.

Appendix A

CONSENT TO PARTICIPATE IN A CLINICAL RESEARCH STUDY

**STUDY TITLE:** **BMT4me: Improving Adherence through mHealth for Pediatric Stem Cell Transplant Patients**

PRINCIPAL INVESTIGATOR: *Dr. Micah Skeens*

CONTACT TELEPHONE NUMBER: *614-722-8958 (8am-4pm, Monday-Friday)*

SUBJECT’S NAME: ____________________________ DATE OF BIRTH: _________________

NOTE: The words “you” and “your child” are used in this consent form. These words refer to the study volunteer whether a child or an adult*.*

| **Key Information About This Study**  The following is a short summary of this study to help you decide whether or not to participate. More detailed information follows later in this form.  The purpose of this study is to evaluate the acceptability of the newly developed mHealth app (BMT4me) and the potential efficacy of a mHealth app on adherence to immunosuppressants in children who have been discharged home during the first 100 days after transplant (HSCT).  Study participation: Subjects will complete several questionnaires about their background, medication taking, and, if in the intervention group, their feelings towards the BMT4me app. These will be completed first at enrollment, then weekly for three months after initial enrollment in the study *or* until their child is weaned off of immunosuppressants. Surveys will be administered via REDCap text link, secure email, and/or during follow-up clinic appointments. Additionally, all participants will be given their own MEMS (Medication Event Monitoring System) Cap device or Medy RPM medication box to administer medications with throughout the duration of the study. All families will receive a $50 gift card at the beginning of the study and another $25 gift card at the end.  Study visits: The study will recruit 50 caregivers of children post-transplant and prior discharge. After informed consent/assent, caregivers will complete baseline assessments before randomization to either the intervention (BMT4me app) or usual care. Both groups will receive a MEMS Cap device on the immunosuppressant medication to accurately measure medication taking. Brief follow-up assessments will occur weekly in both groups. See a more detailed discussion later in this form.  The main risk(s) of the study are loss of confidentially and possibly feeling minor irritation or upset when completing tasks or answering questions, but it may be more likely that you find them a little boring. We believe that there is very little chance that bad things will happen as a result of being in this study. Other risks are listed later in this form.  The benefit(s) of the study are that children of participants assigned to the BMT4me app (intervention) group may miss fewer doses of medication. Both groups may benefit from frequent reporting and check-ins from study staff. We hope to learn ways to improve the app for future use. We will reimburse you for your time at the end of the study.  If you are interested in learning more about this study, please continue reading below. |
| --- |

# 1) INTRODUCTION

We invite you to be in this research study. Using this form as a guide, we will explain the study to you. If you have any questions about the study, please ask. By signing this form, you agree to be in this study. If you do not want to be in this study, all regular and standard medical care will still be available to you here at Nationwide Children’s Hospital. Participation is voluntary. You can leave this study at any time.

You will be given a signed and dated copy of the consent and the assent forms.

# 2) WHERE WILL THE STUDY BE DONE AND HOW MANY SUBJECTS WILL TAKE PART?

This study will be done at Nationwide Children’s Hospital and we hope to enroll 50 participants.

3) WHAT WILL HAPPEN DURING THE STUDY AND HOW LONG WILL IT LAST?

Once you have agreed to be in the study, you will be randomized to receive the BMT4me app or usual care. Randomized means that each subject will be picked by chance, like tossing a coin or drawing straws, to receive either the study intervention (using the BMT4me app to track medications) or the non-intervention (NOT using the BMT4me app to track medications). Each subject has a 50/50 chance of using the BMT4me app and a 50/50 chance of not using the BMT4me app. Regardless of which group you are in, you will receive a MEMS (Medication Event Monitoring System) Cap bottle or Medy Remote Patient Management (RPM) medication box to put your child’s immunosuppressant medication in and will be asked to complete a few baseline measures that take 15-20 minutes. These will ask about your background and your child’s medication taking. If you are randomized to the BMT4me app group, you will have the application installed on your personal cell phone device at no cost. Research staff will conduct a brief tutorial on functions and demonstrate use. You will then enter your child’s immunosuppressant medications into the app to help you remember when to give your child their medications. Your primary discharge nurse will help with this process to make sure your reminders and dosages are correct. All families will complete the study when they reach Day 100 post-transplant or begin to taper off of immunosuppression, whichever is first. Weekly and monthly surveys will be administered via REDCap text link, secure email, and/or during follow-up clinic appointments. At the conclusion of the study, caregivers in the BMT4me app group will complete a measure asking about the usability of the BMT4me app. Finally, caregivers in both groups will be asked to participate in a 15–30 minute. semi-structured interview. A $50 gift card will be provided to all families at enrollment and another $25 at the end of the study. Please note that the digital data we collect from you, which records your usage of the MEMS cap or BMT4me app, will **not** be shared with your provider while you are in the study. We collect this data only for analysis purposes once the study has ended.

4) WHAT ARE THE RISKS OF BEING IN THIS STUDY?

We believe that there is very little chance that bad things will happen as a result of being in this study.

It is possible that you could feel upset when answering questions about your diagnosis or medical treatment, but it may be more likely that you find the questions or feedback process a little boring. If you do find any of the questions upsetting or don’t want to answer a question, you don’t have to, and the study team will be available to discuss this with you further.

Although we will take every precaution, there is a small chance of loss of confidentiality of your study information.

If you are worried about anything while in this study, please call the study team at the telephone number on page 1 of this form.

**5) ARE THERE BENEFITS TO TAKING PART IN THIS STUDY?**

Possible benefits to you might be that children of participants may miss fewer doses of medication and thus suffer from fewer readmissions and/or side effects of missed medications. And, we might learn something that could help others.

8) WHAT ARE THE COSTS AND REIMBURSEMENTS?

For your time and inconvenience, you (study participant) will receive a $50 gift card at enrollment and a $25 gift card at the end of your time in the study. You will be issued a debit card specially designed for clinical research. When a study visit is completed, funds will be approved and automatically loaded onto your card. The funds will be available immediately after being loaded, but it could take up to 1-2 business days. These funds can be used at your discretion. Each participant will be issued one card. If the card is lost or stolen, please call the study coordinator for a replacement card.

Each participant’s name, subject number, address, email address, and cell phone number will be collected by Nationwide Children’s Hospital to issue the debit cards. Debit cards are managed by Greenphire Inc. All information is stored in a secure fashion. The information collected will not be shared with any third parties and will be kept completely confidential.

If you receive $600 or more in a calendar year from participating in research studies, you will be issued a 1099 IRS Form to file with your income taxes.

**9) WHAT HAPPENS IF BEING IN THIS STUDY CAUSES INJURIES?**

We believe that there is very little chance that injuries will happen as a result of being in this study. If you have questions or are worried about your rights as a research volunteer, contact the Nationwide Children’s Hospital Institutional Review Board (IRB), a group that reviews all research at Nationwide Children’s Hospital at (614) 722-2708. Concerns may also be addressed at any time during your participation with the study investigator.

11) WHAT HAPPENS IF I DO NOT FINISH THIS STUDY?

It is your choice to be in this study. You may decide to stop being in this study at any time. If you decide to stop being in this study, call the study team at the number on page 1 of this form to see if there are any medical issues about stopping. If you stop being in the study, there will be no penalty or loss of benefits to which you are otherwise entitled.

If at any time the Principal Investigator believes that this study is not good for you, the study team will contact you about stopping. If the study instructions are not followed, participation in the study may also be stopped. If unexpected medical problems come up, the Principal Investigator or the Sponsor, may decide to stop your participation in the study.

12) OTHER IMPORTANT INFORMATION

If you are an employee of Nationwide Children’s Hospital or the Research Institute at Nationwide Children’s Hospital, your job or performance appraisal will not be affected in any way if you decline to participate or withdraw your consent to participate in this study.

If you are interested, the final study results will be shared with you once they are available. Please provide us with an email or address where we can send these results.

Military personnel should check with their supervisor before accepting payment for participation in this research.

Nationwide Children’s Hospital is a teaching hospital and we are committed to doing research. Doing research will enable us to learn and provide the best care for our patients and families. You may be asked to participate in other research studies in the future. You have the right to decide to participate or decline to participate in any future studies. We will not share your contact information with researchers outside Nationwide Children’s Hospital.

**13) HOW WILL MY STUDY INFORMATION BE KEPT PRIVATE?**

Information collected for this study includes information that can identify you. This is called “protected health information” or PHI. By agreeing to be in this study, you are giving permission to your health care provider to use or disclose (release) your health information that identifies you for the research study described in this form. Information collected is the property of Nationwide Children’s Hospital, its affiliated entities, and/or the sponsor*.*

- PHI that may be used or disclosed will include: Names; Telephone number; Your child’s medical record number and date of diagnosis; Dates of Birth; Discharge Date; E-mail Addresses

**People or Companies authorized to use, disclose, and receive PHI collected or created by this research study:**

- PI and study staff
- The Nationwide Children’s Hospital Institutional Review Board (the committee that reviews all human subject research)
- Nationwide Children’s Hospital internal auditors
- Office for Human Research Protections (OHRP)

Because of the need to give information to these people, absolute confidentiality cannot be guaranteed. Information given to these people may be further disclosed by them and no longer be protected by federal privacy rules.

**Reason(s) why the use or disclosure is being made:** To locate your (child’s) medical records; To contact you in the future

You may decide not to authorize the use and disclosure of your PHI. However, if it is needed for this study, you will not be able to be in this study. If you agree to be in this study and later decide to withdraw your participation, you may withdraw your authorization to use your PHI. This request must be made in writing to the Principal Investigator at Nationwide Children’s Hospital, Center for Biobehavioral Health, NEOB 3^rd^ Fl., Columbus, OH 43205. If you withdraw your authorization, no new PHI may be collected and the PHI already collected may not be used unless it has already been used or is needed to complete the study analysis and reports.

PHI will only be shared with the groups listed above, but if you have a bad outcome or adverse event from being in this study, the study team or other health care providers may need to look at your entire medical records.

The results from this study may be published but your identity will not be revealed.

A copy of this form and other research related health information may be added to your NCH medical record.

The PHI collected or created under this research study will be used or disclosed as needed until the end of the study. The records of this study will be kept for an indefinite period of time and your authorization to use or disclose your PHI will not expire.

There is a risk that someone could get access to the information (data) we have collected about you. If those data suggested something serious about your health, it could be misused. For example, it could be used to make it harder for you to get or keep a job or insurance. The Genetic Information Nondiscrimination Act of 2008 (GINA) says that group and individual health insurers may not use your genetic information to determine whether you are eligible for insurance, how much you have to pay, nor can they request or require that you take a genetic test. We cannot guarantee that this will fully protect you. Your privacy and the confidentiality of your data are very important to us. We will make every effort to protect them.

**Certificate of Confidentiality**

This research is covered by a Certificate of Confidentiality from the National Institutes of Health. This means that the researchers cannot release or use information, documents, or samples that may identify you in any action or suit unless you say it is okay. They also cannot provide them as evidence unless you have agreed. This protection includes federal, state, or local civil, criminal, administrative, legislative, or other proceedings. An example would be a court subpoena.

There are some important things that you need to know. The Certificate DOES NOT stop reporting that federal, state or local laws require. Some examples are laws that require reporting of child or elder abuse, some communicable diseases, and threats to harm yourself or others. The Certificate CANNOT BE USED to stop a sponsoring United States federal or state government agency from checking records or evaluating programs. The Certificate DOES NOT stop disclosures required by the federal Food and Drug Administration (FDA). The Certificate also DOES NOT prevent your information from being used for other research if allowed by federal regulations.

Researchers may release information about you when you say it is okay. For example, you may give them permission to release information to insurers, medical providers or any other persons not connected with the research. The Certificate of Confidentiality does not stop you from willingly releasing information about your involvement in this research. It also does not prevent you from having access to your own information.

**14) USE OF INFORMATION/SAMPLES FOR FUTURE RESEARCH USE**

Information that identifies you may be removed from your study data and your data distributed to other investigators to be used for future research studies without your additional informed consent.

**Future Research Use of Identifiable Information:**

With your permission, we would like to store your identifiable information (including PHI) for future research purposes, and as part of such future research purposes, your identifiable information may be disclosed to people or entities not listed above, such as researchers not involved with this study, government agencies, research foundations, or pharmaceutical or device companies. This future research may or may not be related to your medical problem. This future research may include sensitive information. Any future research projects will be reviewed and approved by an Institutional Review Board which protects the rights, welfare, and safety of human research subjects. If your identifiable information including PHI is used or disclosed in future research studies, absolute confidentiality cannot be guaranteed. Information shared for future research may be shared further with others and no longer be protected by federal privacy rules.

If you decide at any time that you do not want your PHI stored for future research, you must make this request in writing to the Principal Investigator at Nationwide Children’s Hospital, Center for Biobehavioral Health, NEOB 3^rd^ Fl., Columbus, OH 43205Once we receive your written request, we will destroy your PHI. However, if we have already shared your PHI with another individual or entity, we will not be able to destroy any of the PHI that are no longer in our possession.

Nationwide Children’s Hospital retains the right to cease storage and destroy the PHI at any time without sending notice to you or obtaining your consent.

You do not have to agree to use of your PHI for future research in order to be in this study, and your decision will not affect the care you receive from the study doctors or Nationwide Children’s Hospital.

I agree to allow my PHI to be stored and used for future research as described above: (initial)

____ YES ____ NO

**15) WHOM SHOULD I CALL IF I HAVE QUESTIONS OR PROBLEMS?**

If you have questions about anything while on this study or you have been injured by the research, you may contact the Principal Investigator at 614-722-8958, Monday – Friday, between 8am-4pm.

If you have questions, concerns, or complaints about the research; if you have questions about your rights as a research volunteer; if you cannot reach the Principal Investigator; or if you want to call someone else, call (614) 722-2708, Nationwide Children's Hospital Institutional Review Board, (the committee that reviews all research involving human subjects at Nationwide Children’s Hospital).

**Signature Block for Adult Participation  N/A, Pediatric Subject**

Your signature documents your permission to take part in this research.

____________________________________________________ _________________________

Signature of subject Date & Time AM/PM

_____________________________________________________

Printed name of subject

_____________________________________________________ _________________________

Signature of person obtaining consent Date & Time AM/PM

_____________________________________________________

Printed name of person obtaining consent
