# Supplementary material for: Improving medication adherence monitoring and clinical outcomes through mHealth: A randomized controlled trial protocol in pediatric stem cell transplant
Source: PLoS One. 2023 Aug 17;18(8):e0289987. doi: 10.1371/journal.pone.0289987 (PMC10434937; doi:10.1371/journal.pone.0289987)
Supplement: S2 File — (DOCX) [file pone.0289987.s002.docx]

**PROTOCOL TITLE:**

BMT4me: Improving Adherence through mHealth for Pediatric Stem Cell Transplant Patients

**PRINCIPAL INVESTIGATOR:**

Name: Micah Skeens, Ph.D.

Department/Center: Center for Biobehavioral Health

Telephone Number: (614) 722-8958

Email Address: Micah.Skeens@nationwidechildrens.org

**VERSION NUMBER/DATE:**

*Version #1 02/10/2022*

**REVISION HISTORY**

| **Revision #** | **Version Date** | **Summary of Changes** | **Consent Change?** |
| --- | --- | --- | --- |
| 1 | 08-16-2022 | Details added about online REDCap consent (eConsent) | No |
| 2 | 8-24-2022 | Section 6.0 “Procedures Involved”, subitem 6.4 “Data Collected” was updated to include three new participant-facing measures. | No |
| 3 | 9-6-2022 | Details added about changing eligible age range to 2 years of age to 18 years of age. Compensation at time 1 is also changed to $50. Duplicate of the Caregiver Satisfaction instrument has been removed. | No |
| 4 | 10-06-2022 | Changing age range to 0-18 years of age. Included information regarding REDCap text links and Medy Remote Patient Management medication box. | Yes |
| 5 | 01-16-2023 | Changing age range to 0-21 years old.  Adding that patients must be residing with a primary caregiver before enrollment to the study.  Increasing sample size to 50 participants. | Yes |
| 6 | 03-15-2023 | Changing verbiage regarding end of study criteria (i.e., changing from “begin taper” to “complete taper”). | No |
| 7 | 04-27-2023 | Updating inclusion criteria to include caregivers of children receiving autologous hematopoietic stem cell transplants. Including the PedsQL measure for adolescents ages 18-25. Converted “MEMS Caps” and “Medy boxes” to more general “electronic adherence monitoring devices” language. Corrected grammatical errors. | Yes |

# Study Summary

| **Study Title** | BMT4me: Improving Adherence through mHealth for Pediatric Stem Cell Transplant Patients |
| --- | --- |
| **Study Design** | Prospective longitudinal pilot RCT |
| **Primary Objective** | The primary objective is to evaluate the acceptability of the newly developed mHealth app (BMT4me) and the feasibility of enrolling and retaining 50 caregivers of children in the acute phase post-HSCT in a small-scale RCT |
| **Secondary Objective(s)** | Evaluate the potential efficacy of a mHealth app on adherence to immunosuppressant and anti-infective medications in children who have been discharged home during the acute phase post-HSCT |
| **Research Intervention(s)/ Investigational Agent(s)** | N/A |
| **IND/IDE #** | N/A |
| **Study Population** | Primary caregivers of children undergoing allogenic or autologous HSCT at Nationwide Children’s Hospital (NCH). |
| **Sample Size** | 50 caregivers |
| **Study Duration for individual participants** | 100 days (~3.5 months) or until the weaning of immunosuppressants (whichever is first). |
| **Study Specific Abbreviations/ Definitions** | NCH: Nationwide Children’s Hospital; HSCT: Allogeneic Hematopoietic Stem Cell Transplant; GVHD: Graft Versus Host Disease; BMT4me: adherence application and “personal assistant” for caregivers of children receiving HSCT, post-HSCT, or discharged after receiving HSCT.; MEMS; Medication Event Monitoring System; Medy RPM (remote patient management); |

# Objectives

- 1. The goal of the study is to conduct a pilot RCT (N = 50) to assess feasibility, acceptability, and preliminary efficacy of the mHealth intervention on adherence to immunosuppressant or anti-infective medications (primary outcome) and key clinical outcomes (secondary outcome) such as graft vs. host disease and readmissions relative to usual care.
  2. Aim 1: To evaluate the acceptability of the newly developed mHealth app and the feasibility of enrolling and retaining 50 caregivers of children in the acute phase post-HSCT in a small-scale RCT.
     - Hypothesis 1: Caregivers will report above average acceptability (> 68%), and > 75% of participants will enroll and complete all study-related assessments.

Aim 2: Evaluate the potential efficacy of a mHealth app on adherence to immunosuppressants or anti-infectives in children who have been discharged home during the acute phase post-HSCT.

- - - Hypothesis 2a: Patients with caregivers randomized to the mHealth app will have higher adherence frequency (Medication Possession Ration and self-report) than the usual care group.
    - Hypothesis 2b: Patients with caregivers randomized to the app will have less GVHD and fewer readmissions than the usual care group.

Exploratory Aims: (1) Explore moderators (e.g., sex, age, socioeconomic status) and the most common mediator, forgetfulness, in the association between the mHealth app and adherence. (2) Explore the potential utility of the Medication Level Variable Index (MLVI) in relation to other measures of adherence (e.g., MPR, self-report).

# Background

- 1. In the United States, poor adherence accounts for up to 70% of all medication-related hospital admissions, resulting in $100 billion in healthcare costs annually Adherence rates have been reported as low as 0% in pediatric patients. Reasons for non-adherence are multifactorial. The most important determinants of non-adherence are consistently documented as complexity and duration of treatment regimens, as well as forgetfulness. Thus, children undergoing difficult hematopoietic stem cell transplants (HSCT) that require medication indefinitely are at high risk for medication non-adherence.
  2. Only 4 published studies exist regarding adherence in pediatric HSCT. None address adherence to immunosuppressant medication, nor are they RCTs. Second, the complexity of most interventions for adherence is counter to the geographic, resource, and time constraints families of chronically ill children face. Adherence interventions based on conventional behavior theory have been cumbersome for families already stressed due to chronic illness. BE design is a significant paradigm shift to a simpler, less onerous approach that can engage those patients and families that would otherwise forego complicated adherence interventions. Although mHealth adherence apps are a widely available, simple, and innovative approach to addressing these problems, a third gap relates to poor usability. For example, a recent review of pediatric adherence apps found that none identified individual barriers to adherence, and nearly all were designed for adults. Thus, there is an urgent need to develop and evaluate innovative, accessible, and evidence-based approached to adherence among children receiving HSCT to prevent morbidity and mortality from GVHD.
  3. The impact of non-adherence on clinical outcomes is largely unknown in pediatric HSCT. Poor adherence is generally associated with adverse outcomes, including complications, hospital admissions, and even death. The societal burden of cancer care and HSCT is substantial and likely to increase based on the growing number of transplants each year. Clinicians and researchers have focused on GVHD prevention to minimize unnecessary treatment-related deaths. Acute GVHD develops in the first 100 days post-transplant. Children that develop acute GVHD have a 30% to 50% chance of survival. Morbidity and mortality due to GVHD can be decreased through prophylactic use of immunosuppressants. Although these medications are costly and produce unpleasant side effects, adherence is critical to decrease complications, reduce readmissions, and ultimately increase quality of life and survival.

Adherence is complex, but ultimately, the final common pathway to adherence is human behavior. In pediatrics, adherence is largely dependent on parents. As the primary caregivers, they are responsible for ensuring children receive the prescribed therapy correctly. In a high-risk HSCT population, caregivers are isolated with their child due to infection risk and must manage challenging treatment regimens at home, often with limited time and support. Complex behavioral interventions, typically employed to address adherence, are difficult to deliver and manage in the context of these daily tasks. Alternatively, behavioral economics (BE) theory suggests that small “nudges” can produce and sustain behavior change. A BE approach is a significant paradigm shift and assumes decision-making can be influenced through low-intensity interventions to lead patients to optimal choices. Improved adherence to medication and exercise programs using BE designed interventions in adults have been positive. Within pediatrics, BE has been successful in reducing childhood obesity, increasing vaccination rates, and improving adherence rates to infant HIV medications.

# Study Endpoints

- 1. Study endpoints are to evaluate (1) the acceptability of the newly developed mHealth app and the feasibility of enrolling and retaining 50 caregivers of children in the acute phase post-HSCT (i.e., first 3 months post-discharge) and (2) the potential efficacy of the mHealth app on adherence to immunosuppressants or anti-infectives in children who have been discharged during the acute phase post-HSCT.
     - N/A

# Study Intervention/Investigational Agent

- 1. Description:

1. **mHealth “BMT4me” App**. After caregivers of HSCT children have provided informed consent via pen and paper or electronically on REDCap (using REDCap electronic signing module) for the RCT, they will complete baseline assessments before randomization to either the intervention (mHealth “BMT4me” app) or usual care. The BMT4me app was designed by AWRI RISI developers and has been through multiple phases of stakeholder testing. The app is a virtual assistant for caregivers, allowing for medication record keeping, medication and refill reminders, symptom tracking, and a word and picture journal. All data is secured on the individuals password protected phone.
   1. Drug/Device Handling: If the research involves drugs or device, describe your plans to store, handle, and administer those drugs or devices so that they will be used only on subjects and be used only by authorized investigators.
      - N/A
      - If the control of the drugs or devices used in this protocol will be accomplished by following an established, approved organizational SOP (e.g., Research Pharmacy SOP for the Control of Investigational Drugs, etc.), please reference that SOP in this section.
        - N/A
   2. If the drug is investigational (has an IND) or the device has an IDE or a claim of abbreviated IDE (non-significant risk device), include the following information:
      - N/A
      - Identify the holder of the IND/IDE/Abbreviated IDE.
        - N/A
      - Explain procedures followed to comply with sponsor requirements for FDA regulated research for the following:
        - N/A

|  | ***Applicable to:*** | | |
| --- | --- | --- | --- |
| ***FDA Regulation*** | ***IND Studies*** | ***IDE studies*** | ***Abbreviated IDE studies*** |
| ***21 CFR 11*** | ***X*** | ***X*** |  |
| ***21 CFR 54*** | ***X*** | ***X*** |  |
| ***21 CFR 210*** | ***X*** |  |  |
| ***21 CFR 211*** | ***X*** |  |  |
| ***21 CFR 312*** | ***X*** |  |  |
| ***21 CFR 812*** |  | ***X*** | ***X*** |
| ***21 CFR 820*** |  | ***X*** |  |

# Procedures Involved*

- 1. Describe and explain the study design.
     - The study will recruit 50 caregivers of children post allogeneic or autologous HSCT for a pilot RCT prior to discharge. Study team members will go over consent with participants; if they agree to participate in the study, participants will be provided the consent form via pen and paper or online via REDCap and will complete it electronically. REDCap has a digital signature module that users can use with a mouse or touch screen to sign relevant parts of the consent document. After informed consent/assent for the RCT, caregivers will be randomized to either the intervention (mHealth app) or usual care. The randomization sequence will be based on a design with blocks of four or six, chosen randomly within the sequence with equal probability. The randomization module in REDCap will be used, and the randomization sequence will be maintained by the statistician. Randomly varying block size reduces the change that research staff will guess the next group assignment, minimizing unconscious bias. Reasons for non-participation and dropout will be tracked. Both groups will receive standard education at discharge with regards to medication, as well as an electronic adherence monitoring device to put their pill or liquid immunosuppressant or anti-infective medication in. These electronic monitoring devices will allow for accurate measures of medication adherence. Caregivers assigned to the intervention group will also have the mHealth adherence app downloaded onto their personal device.

Brief follow-up assessments will occur weekly in both groups. These weekly assessments will be sent to families via REDCap text link, secure email, and/or in clinic during follow-up appointments.

- 1. Provide a description of all research procedures being performed and when they are performed, including procedures being performed to monitor subjects for safety or minimize risks.
     - Rigor and reproducibility will be ensured through a rigorous randomized controlled design; a highly manualized approach to procedures for recruitment and data collection; multiple methods (qualitative and quantitative); standardized measures with strong psychometric properties; centralized/electronic data entry/management, and rigorous statistical tests of proposed hypotheses. Dr. Skeens will monitor the study to ensure intervention fidelity and adherence to regulatory and data management requirements. Interventionists will be trained, and the sessions will be audiotaped and double-coded by Drs. Skeens and Gerhardt to ensure fidelity. The team will discuss recruitment and protocol activities, including intervention fidelity, during regular lab meetings.

Primary caregivers in both groups who consent (via pen and paper or electronically, whichever they prefer) to participate will complete baseline measures prior to discharge from the hospital. Families will be randomized (as described above) to receive the mHealth app or usual care. Caregivers randomized to the mHealth app will have the application installed on their personal cell phone device at no cost. Research staff will conduct a brief tutorial on functions and demonstrate use, these sessions will be audio-recorded (treatment fidelity). The caregiver will add immunosuppressants or anti-infectives and the schedule for administration with oversight by the primary discharge nurse to ensure accuracy. Accuracy of medications within the app will be verified and recorded at each visit (fidelity check). The following data will be recorded on all patients; baseline medical information (i.e., diagnosis, age at diagnosis, treatment history, medications)and weekly adherence measures. Serum immunosuppression assays will be collected as an exploratory aim in children who received allogenic transplants. Monthly electronic adherence monitoring device and clinical outcomes data will also be collected in both groups. Families will complete the study when they reach Day 100 or complete taper from immunosuppression, whichever is first. At the conclusion of the study, caregivers in the mHealth app arm will complete the System Usability Scale. Caregivers in both arms will participate in a 15–30 minute semi-structured interview addressing: 1) experience with adherence post-transplant and participating in the trial (e.g., benefit, burden, barriers, satisfaction) and 2) Caregivers in the app arm will be asked to share any suggested changes to the application. A $50 gift card will be provided to all families at enrollment and another $25 at the end of the study

- 1. Describe:
     - Procedures performed to lessen the probability or magnitude of risks. Privacy concerns will be addressed throughout the conduct of the study. Specifically:

1. All data will be identified by subject numbers and forms that must contain identifying information (consent forms, recruitment records) will be stored separately from information identified by ID number. All data will be stored in secured file space in the Center for Biobehavioral Health. Tracking sheets that link study ID numbers and identifiers will be stored on a secure server and only be accessible to the PI and staff involved in recruitment or specific data management tasks.
2. Participants will be informed through the written or electronic consent process that their data will be kept private. Exceptions to confidentiality related to child safety are disclosed at that time.
3. Dr. Skeens will monitor the study to ensure intervention fidelity and adherence to regulatory and data management requirements. The team will discuss recruitment and protocol activities, including intervention fidelity, during weekly team meetings
   - - All drugs and devices used in the research and the purpose of their use, and their regulatory approval status. N/A
     - The source records that will be used to collect data about subjects. (Attach all surveys, scripts, and data collection forms.) Complete (see eIRB).
   1. What data will be collected during the study and how that data will be obtained.

- Data will be collected via self-report of caregivers. Additional relevant clinical information will be reviewed and extracted via electronic medical records. Finally, data will be collected via the BMT4me app and/or electronic adherence monitoring device, depending on which group they are randomized to. All digital data collected via the electronic adherence monitoring devices and/or the BMT4me app regarding participant usage will **not** be shared with providers while the study is active.
  - - - Demographic Data Form. The caregiver will report on basic background characteristics including parent and child age, sex, race, ethnicity, education level, and family income.
      - Posttransplant Perception Survey. The posttransplant perception survey is a self-report clinical assessment tool adapted from the kidney transplant population for this trial. The tool contains 4 items asking participants to report on their views about their child’s health and perceptions post-transplant. Items are rated on a 5-point Likert scale.
      - Barrier Assessment Tool (BAT)- Caregiver. The Barrier Assessment Tool (BAT) is a self-report clinical assessment tool. The tool contains 14 commonly endorsed barriers with a checkbox next to each item. Domains include logistical issues (e.g., forgetting, inconvenience), ingestion difficulties (e.g., swallowing, taste), efficacy (e.g., feel I don’t need it), financial difficulties, regimen characteristics (e.g., too many medications, side effects), and patient-specific issues (e.g., refusal by child, embarrassment). A sum total of the number of barriers is calculated, as well as concordance between caregiver and patient report. In the feasibility study, the average concordance between caregiver and patient report of each barrier (n=48) was 0.299. The total barrier score will be used. A sum total of 0 to 14 could be calculated, with higher scores indicating more barriers.
      - Caregiver Satisfaction. Satisfaction will be assessed via semi-structured interviews and an electronic version of the Caregiver Satisfaction Questionnaire with caregivers. Caregivers will be asked for feedback regarding participation in the intervention, benefit, burden, barriers, suggested modifications, and overall satisfaction. Suggested modifications to the app and advice to the healthcare team will also be solicited. Due to the qualitative nature of the interview, caregiver responses cannot be scored but will be coded for themes. Questions on the Caregiver Satisfaction Questionnaire are scored on a 1 to 4 Likert scale with higher total scores indicating higher caregiver satisfaction.
      - Medication Possession Ratio (MPR). MPR is a standard adherence measure that uses pharmacy refill records to calculate adherence using the sum of the days’ supply obtained between the first pharmacy fill and the last fill divided by the total number of days. Higher scores indicate better adherence. The number of refills increases accuracy of the adherence estimate.
      - Electronic adherence monitoring device. Electronic adherence monitoring devices collect date and timestamped data regarding medication taking each time the device is opened. Openings and closings are typically recorded via a near field communication (NFC) reader or micro-electronic circuit. Data can be downloaded from using cloud or computer-based software. This data will be collected from participants at each study visit. An adherence percentage will be calculated by dividing the number of doses taken by the number of doses prescribed for each day. All of the electronic monitors have been independently tested for accuracy. Data collected is uploaded to a secure cloudbase software or can be downloaded
      - ^55^
      - Medication Adherence Measure (MAM). The MAM is semi-structured interview specific to pediatrics, conducted with the parent, to obtain an individual score in each module. The score is represented in percentages of the number of required doses. A total summary score can be calculated across all medications, as well as separately. This allows for quantification of the degree of adherence on a continuum. MAM has demonstrated adequate convergent validity with MEMs caps (r =−.40, p < .05).
      - Pediatric Quality of Life Inventory (PedsQL) version 4.0. Parents will complete the Pediatric Quality of Life Inventory (PedsQL) every three weeks (at week 3, week 6, and week 9). The frequency of 23 problems in 4 domains (i.e., physical, emotional, social, school) are rated on a 3 or 5-point scale. Versions are based on child age: (a) 5-7, (b) 8-12, (c) 13-18 years old, and (d) 18-25.
      - System Usability Scale (SUS). The SUS is a 10-item questionnaire routinely used to evaluate the functionality and acceptability of mHealth apps. Items are rated on a 5-point scale and scores range from 0 to 100. Reliability (0.91) and validity (.81 correlation with 7- point scale of “user friendliness”) have been well established. A score of > 68% is considered above average.
      - Medication Level Variability Index (MLVI). The MLVI is the calculation of the standard deviation of serum assays of immunosuppressants that has shown to correlate with adherence and clinical outcomes in the solid organ transplant population. Immunosuppressant serum assays are collected weekly during the acute phase. A calculation of the degree of variation among levels will be formulated.
      - Graft vs. Host Disease (GVHD). GVHD will be assessed on the international standard acute GVHD grading and staging scale. Provider grading will be per organ system on a 1-4 scale, with an overall score given weekly.
      - Readmissions. Readmission rates will be determined by the number of admissions requiring greater than a 24-hour stay within the first 100 days after discharge. Reason for readmission will be recorded and based on the EMR discharge diagnosis. In addition, a sub-analysis of readmissions within the first 30 days after initial discharge post-HSCT will be completed.
  1. If there are plans for long-term follow-up (once all research related procedures are complete), what data will be collected during this period.
     - N/A
  2. For Humanitarian Use Device (HUD) uses provide a description of the device, a summary of how you propose to use the device, including a description of any screening procedures, the HUD procedure, and any patient follow-up visits, tests or procedures.
     - N/A

# Data and Specimen Banking*

- 1. If data or specimens will be banked for future use, describe where the specimens will be stored, how long they will be stored, how the specimens will be accessed, and who will have access to the specimens.
     - N/A
  2. List the data to be stored or associated with each specimen.
     - N/A
  3. Describe the procedures to release data or specimens, including: the process to request a release, approvals required for release, who can obtain data or specimens, and the data to be provided with specimens.
     - N/A

# Sharing of Results with Subjects*

- 1. Describe whether results (study results or individual subject results, such as results of investigational diagnostic tests, genetic tests, or incidental findings) will be shared with subjects or others (e.g., the subject’s primary care physicians) and if so, describe how the results will be shared.
     - As a single site pilot study, we do not intend on sharing study results with subjects or others (e.g., physicians, care team, etc.).

# Study Timelines*

- 1. Describe:
     - The duration of an individual subject’s participation in the study.
     - Participants will be recruited in the acute outpatient phase (first 100 days) post-HSCT from their stem cell transplant unit prior to discharge and will then be enrolled for three and a half months.The duration anticipated to enroll all study subjects.
       - Estimates of potential recruitment/retention rates are based on cancer registry data and strong documented recruitment experience at NCH. With approximately 80 transplants annually at NCH, we will have ample eligible families to approach. The goal is to enroll at least 50 families over 2.5 years. We anticipate 85% mothers as primary caregivers, and children will be equally distributed by sex.
     - The estimated date for the investigators to complete this study (complete primary analyses)
       - Data collection will cease when accrual goals are met. It is anticipated this will be 2.5 years. Primary analysis will occur in the second half of Year 3. With the entire study and primary analysis complete within 3 years.

# Inclusion and Exclusion Criteria*

- 1. Describe how individuals will be screened for eligibility.
     - Caregivers of children who received HSCT will be recruited from the inpatient HSCT unit clinic rosters based on the inclusion and exclusion criteria described in subsection 10.2. All children admitted and scheduled for HSCTs will also be screened for eligibility and recruited prior to discharge.
  2. Describe the criteria that define who will be included or excluded in your final study sample.
     - Children of caregivers must be: a) 0 to 21 years of age; b) receiving immunosuppression for an allogeneic transplant or anti-infective for an autologous transplant; c) discharged prior to Day 100 or immunosuppression taper; d) residing with the primary caregiver that enrolls on study. Primary caregivers must be: e) English-speaking; and f) have an iOS or Android capable cellular device. Caregivers will be excluded if children have a documented developmental delay.
  3. Indicate specifically whether you will include or exclude each of the following special populations: (You may not include members of the above populations as subjects in your research unless you indicate this in your inclusion criteria.)
     - EXCLUDE: Adults unable to consent
     - INCLUDE: Individuals who are not yet adults (children, teenagers)

# EXCLUDE: Prisoners Vulnerable Populations*

- 1. If the research involves individuals who are vulnerable to coercion or undue influence, describe additional safeguards included to protect their rights and welfare.
     - N/A
     - If the research involves pregnant women, review “CHECKLIST: Pregnant Women (HRP-412)” to ensure that you have provided sufficient information.
       - N/A
     - If the research involves neonates of uncertain viability or non-viable neonates, review “CHECKLIST: Neonates (HRP-413)” or “HRP-414 – CHECKLIST: Neonates of Uncertain Viability (HRP-414)” to ensure that you have provided sufficient information.
       - N/A
     - If the research involves prisoners, review “CHECKLIST: Prisoners (HRP-415)” to ensure that you have provided sufficient information.
       - N/A
     - If the research involves persons who have not attained the legal age for consent to treatments or procedures involved in the research (“children”), review the “CHECKLIST: Children (HRP-416)” to ensure that you have provided sufficient information.
       - N/A
     - If the research involves cognitively impaired adults, review “CHECKLIST: Cognitively Impaired Adults (HRP-417)” to ensure that you have provided sufficient information.
       - N/A

# Local Number of Subjects

- 1. Indicate the total number of subjects to be accrued locally.
     - 50 caregivers of children post-HSCT will be collected from Nationwide Children’s Hospital.
  2. If applicable, distinguish between the number of subjects who are expected to be enrolled and screened, and the number of subjects needed to complete the research procedures (i.e., numbers of subjects excluding screen failures.)
     - N/A; Pilot project

# Recruitment Methods

- 1. Describe when, where, and how potential subjects will be recruited.
     - Eligible caregivers will be invited by trained research staff at Nationwide Children’s Hospital, to participate in the study immediately prior to discharge on the inpatient HSCT floor.
  2. Describe the source of subjects.
     - Potentially eligible participants will be identified through accessing clinic schedules and assessing for inclusion/exclusion criteria
  3. Describe the methods that will be used to identify potential subjects.
     - Potentially eligible participants, if eligible, will be introduced and asked to participate by a research team member before their child is discharged from the inpatient HSCT floor.
  4. Describe materials that will be used to recruit subjects. (Attach copies of these documents with the application. For advertisements, attach the final copy of printed advertisements. When advertisements are taped for broadcast, attach the final audio/video tape. You may submit the wording of the advertisement prior to taping to preclude re-taping because of inappropriate wording, provided the IRB reviews the final audio/video tape.)
     - Trained research staff will approach caregivers who meet eligibility prior to the child’s discharge. This initial contact will introduce the study using a pre-determined script. The script study staff reads to enroll a participant and their caregiver will explicitly state the purpose of the study, timeline, that participation is voluntary, and that there is no penalty for declining participation. If they agree, study staff will proceed with formal IRB-approved consent documents (obtained with pen and paper or electronically on an iPad via an electronic signature module on REDCap, whatever the participant prefers). Once consented, instructions & measures will be reviewed with the caregiver prior to beginning the study visit.
  5. Describe the amount and timing of any payments to subjects.
     - A $50 gift card will be provided to all families at enrollment and another $25 at the end of the study, for a total of $75 in subject compensation.

# Withdrawal of Subjects*

- 1. Describe anticipated circumstances under which subjects will be withdrawn from the research without their consent.
     - In the very unlikely event a participant becomes overly distressed from the questions, the examiner will discontinue the protocol and refer the family to psychosocial services if desired.
  2. Describe any procedures for orderly termination.
     - Participants are free to withdraw from the study at any time upon verbal or written request.
  3. Describe procedures that will be followed when subjects withdraw from the research, including partial withdrawal from procedures with continued data collection.
     - Existing data will remain in the study database. If a participant wishes to have all research being conducted on his/her data stopped, the data will not be included in the analysis.

# Risks to Subjects*

- 1. List the reasonably foreseeable risks, discomforts, hazards, or inconveniences to the subjects related the subjects’ participation in the research. Include as may be useful for the IRB’s consideration, a description of the probability, magnitude, duration, and reversibility of the risks. Consider physical, psychological, social, legal, and economic risks.
     - There are no anticipated risks to participating. However, in the very unlikely event a participant becomes overly distressed from the questions, the examiner will discontinue the protocol and refer the family to psychosocial services if desired.
  2. If applicable, indicate which procedures may have risks to the subjects that are currently unforeseeable.
     - N/A
  3. If applicable, indicate which procedures may have risks to an embryo or fetus should the subject be or become pregnant.
     - N/A
  4. If applicable, describe risks to others who are not subjects.
     - N/A

# Potential Benefits to Subjects*

- 1. Describe the potential benefits that individual subjects may experience from taking part in the research. Include as may be useful for the IRB’s consideration, the probability, magnitude, and duration of the potential benefits.
     - Children of participants may miss fewer doses of medications, thus becoming more adherent and suffering from fewer readmissions and/or side effects of missed medications, due to closer monitoring (electronic adherence devices)) and/or reminders from the BMT4me app.
  2. Indicate if there is no direct benefit. Do not include benefits to society or others.
     - N/A

# Data Management* and Confidentiality

- 1. Describe the data analysis plan, including any statistical procedures or power analysis.
     - **Qualitative analyses**. Interviews will be audio-taped and transcribed verbatim for content analysis using the constant comparison method, by at least 2 independent, trained, doctoral level coders. In brief, we will begin with immersion (i.e., repeatedly reading a subset of transcripts), cluster similar ideas to inform preliminary categories, review and revise coding schemes, apply the coding scheme to a second subset of transcripts, revise themes, and repeat this process until reaching saturation and consensus. Member checking will be completed with a subsample to obtain family input on thematic codes as a final validity check, and frequency counts of final themes will be obtained.
     - **Quantitative analyses.** During the passive use observation period, passive data modules will capture phone activity and caregivers’ application use (e.g., time/date, duration of use). Descriptive statistics will be used to analyze phone activity. Correlation analysis will be used to investigate use behavior over time. Acceptability will be assessed by averaging total scores from the System usability scale. Consistent with the literature,^52^ scores > 68% on the SUS will be considered acceptable.
     - **Power.** Given this is a pilot RCT, the overall goal is to examine preliminary efficacy and establish reliable effect sizes to inform a larger multi-site RCT that will be sufficiently powered. Thus, the sample of 50 (25 intervention, 25 usual care only) caregivers. The potential efficacy of the intervention will be examined in the R00 phase using an independent samples t-test where the primary outcome is the proportion of adherence. If adherence is substantially non-normal, efficacy will be examined using an analogous logistic regression model. Because the nature of the study is exploratory rather than confirmatory, the objective of the analysis is effect size estimation rather than formal hypothesis testing, and threats to power (e.g., participant attrition due to patient death, early taper) are not a primary concern. We will compute effect sizes (e.g., a standardized mean difference) for the randomized group comparison to use for our future work assessing the efficacy of the intervention.
  2. Describe the steps that will be taken to secure the data (e.g., training, authorization of access, password protection, encryption, physical controls, certificates of confidentiality, and separation of identifiers and data) during storage, use, and transmission.
     - A system for monitoring recruitment, data collection, and general conduct of the study will include detailed training, weekly lab meetings, and secured collection and storage of data. All data will be checked in real time to ensure completeness, and if items are not completed, the reason will be documented. Any paper copies will be marked with ID numbers and stored in a secure location. All data will be entered into a database by a trained research assistant and verified by the PI. All data files will be maintained in REDCap (Research Electronic Data Capture), a central electronic database system securely stored at the Research Institute at NCH. The master file will be maintained in the PI’s password-protected computer
  3. Describe any procedures that will be used for quality control of collected data.
     - Some data will be collected using paper documents, then manually entered in an electronic database using REDCap. These data points will be entered twice, each time by a different staff member. A third staff member will then conduct a data comparison check, to ensure all data was accurately entered electronically. Study staff will also regularly review and address any protocol deviations, if needed.

Additionally, interviews will be audio-taped and transcribed verbatim for content analysis using the constant comparison method, by at least 2 independent, trained, doctoral level coders. Member checking will be completed with a subsample to obtain family input on thematic codes as a final validity check, and frequency counts of final themes will be obtained.

- 1. Describe how data or specimens will be handled study-wide:
     - What information will be included in that data or associated with the specimens?
       - N/A
     - Where and how data or specimens will be stored?
       - Data will be stored in locked cabinets and/or secure hospital servers which are only accessible by research staff.
     - How long the data or specimens will be stored?
       - Data will be stored until research is completed and may be stored longer according to NCH regulations.
     - Who will have access to the data or specimens?
       - Research staff only.
     - Who is responsible for receipt or transmission of the data or specimens?
       - The Primary Investigator.
     - How data or specimens will be transported?
       - No data from NCH will be transported.

# Provisions to Monitor the Data to Ensure the Safety of Subjects*

This section is required when research involves more than Minimal Risk to subjects.

This study does not involve greater than minimal risk.

- 1. Describe:
     - The plan to periodically evaluate the data collected regarding both harms and benefits to determine whether subjects remain safe. The plan might include establishing a data monitoring committee and a plan for reporting data monitoring committee findings to the IRB and the sponsor.
       - N/A
     - What data are reviewed, including safety data, untoward events, and efficacy data.
       - N/A
     - How the safety information will be collected (e.g., with case report forms, at study visits, by telephone calls with participants).
       - N/A
     - The frequency of data collection, including when safety data collection starts.
       - N/A
     - Who will review the data.
       - N/A
     - The frequency or periodicity of review of cumulative data.
       - N/A
     - The statistical tests for analyzing the safety data to determine whether harm is occurring.
       - N/A
     - Any conditions that trigger an immediate suspension of the research.
       - N/A

# Provisions to Protect the Privacy Interests of Subjects

- 1. Describe the steps that will be taken to protect subjects’ privacy interests. “Privacy interest” refers to a person’s desire to place limits on whom they interact or whom they provide personal information.
     - Privacy will be addressed when caregivers are first approached before discharge. Caregivers who agree to participate will sign informed consent forms (paper and pen or electronically via electronic signature on REDCap, whichever they prefer) as appropriate for their age.
  2. Describe what steps you will take to make the subjects feel at ease with the research situation in terms of the questions being asked and the procedures being performed. “At ease” does not refer to physical discomfort, but the sense of intrusiveness a subject might experience in response to questions, examinations, and procedures.
     - The study involves minimal risk and all measures have been used routinely in the field and by this research team for many years. Study staff are trained to work with families, establish rapport, and monitor for any concerns. Parents will provide informed consent via paper and pen or electronically on REDCap (whichever they prefer) and will be informed at each phase that participation is voluntary. All participants will be informed that they may skip any items that they find distressing.
  3. Indicate how the research team is permitted to access any sources of information about the subjects.
     - The research team will obtain written or electronic consent to access and data and medical records for child participants via hospital approved Epic accounts. As required, all team members will be on the IRB and trained in research ethics prior to involvement with any participants or data.

# Compensation for Research-Related Injury

- 1. If the research involves more than Minimal Risk to subjects, describe the available compensation in the event of research related injury.
     - N/A
  2. Provide a copy of contract language, if any, relevant to compensation for research-related injury.
     - N/A

# Economic Burden to Subjects

- 1. Describe any costs that subjects may be responsible for because of participation in the research.
     - There are no costs to participants.

# Consent Process

- 1. Indicate whether you will you be obtaining consent, and if so describe:
     - Where will the consent process take place?
       - Caregivers who agree to participate will be guided through the HRP-502 consent form. The consent will be completed before starting the study, via pen and paper or on an online REDCap form (REDCap has a digital signature module that users can use a mouse or touch screen to sign the document. Informed consent forms will be signed electronically a few days prior to discharge in the HSCT clinic.
     - Any waiting period available between informing the prospective subject and obtaining the consent.
       - No.
     - Any process to ensure ongoing consent.
       - Families are free to drop out of the study at any time. There is no specific process to reconsent participants.
     - Whether you will be following “SOP: Informed Consent Process for Research (HRP-090).” If not, describe: We will be following this process.
       - The role of the individuals listed in the application as being involved in the consent process.
         1. N/A
       - The time that will be devoted to the consent discussion.
         1. N/A
       - Steps that will be taken to minimize the possibility of coercion or undue influence.
         1. N/A
       - Steps that will be taken to ensure the subjects’ understanding.
         1. N/A

**Non-English Speaking Subjects – if known, skip if not known**

- - - Indicate what language(s) other than English are understood by prospective subjects or representatives.
      - N/A
    - If subjects who do not speak English will be enrolled, describe the process to ensure that the oral and written information provided to those subjects will be in that language. Indicate the language that will be used by those obtaining consent.
      - N/A

**Waiver or Alteration of Consent Process (consent will not be obtained, required information will not be disclosed, or the research involves deception)**

- - - Review the “CHECKLIST: Waiver or Alteration of Consent Process (HRP-410)” to ensure you have provided sufficient information for the IRB to make these determinations.
      - N/A
    - If the research involves a waiver the consent process for planned emergency research, please review the “CHECKLIST: Waiver of Consent for Emergency Research (HRP-419)” to ensure you have provided sufficient information for the IRB to make these determinations.
      - N/A

**Waiver of Written Documentation of Consent (verbal)**

- Review the CHECKLIST: Waiver of Written Documentation of Consent Process (HRP-411) to ensure you have provided sufficient information for the IRB to make these determinations.

**Subjects who are not yet adults (infants, children, teenagers)**

- - - Describe the criteria that will be used to determine whether a prospective subject has not attained the legal age for consent to treatments or procedures involved in the research under the applicable law of the jurisdiction in which the research will be conducted. (E.g., individuals under the age of 18 years.)
      - For research conducted in the state, review “SOP: Legally Authorized Representatives, Children, and Guardians (HRP-013)” to be aware of which individuals in the state meet the definition of “children.”
        1. N/A; Only caregivers of children ages 0-21 will be recruited and consented (electronically via REDCap or with paper and pen) to participate in this study- no children will be enrolled.
      - For research conducted outside of the state, provide information that describes which persons have not attained the legal age for consent to treatments or procedures involved the research, under the applicable law of the jurisdiction in which research will be conducted. One method of obtaining this information is to have a legal counsel or authority review your protocol along the definition of “children” in “SOP: Legally Authorized Representatives, Children, and Guardians (HRP-013).”
        1. N/A; Only caregivers of children ages 0-21 will be recruited and consented (electronically or with pen and paper) to participate in this study- no children will be enrolled.
    - Describe whether parental permission will be obtained from:
      - Both parents unless one parent is deceased, unknown, incompetent, or not reasonably available, or when only one parent has legal responsibility for the care and custody of the child.
        1. N/A; Only caregivers of children ages 0-21 will be recruited and consented (electronically or with pen and paper) to participate in this study- no children will be enrolled.
      - One parent even if the other parent is alive, known, competent, reasonably available, and shares legal responsibility for the care and custody of the child.
        1. N/A; Only caregivers of children ages 0-21 will be recruited and consented (electronically or with pen and paper) to participate in this study- no children will be enrolled.

Describe whether permission will be obtained from individuals other than parents, and if so, who will be allowed to provide permission. Describe the process used to determine these individuals’ authority to consent to each child’s general medical care.

Caregivers with legal guardianship to consent to medical treatment will also be permitted to consent (electronically or with pen and paper) to this study via digital signature on REDCap.

- - - Indicate whether assent will be obtained from all, some, or none of the children. If assent will be obtained from some children, indicate which children will be required to assent.
      - N/A; Only caregivers of children ages 0-21 will be recruited and consented (electronically or with pen and paper) to participate in this study- no children will be enrolled.
    - When assent of children is obtained describe whether and how it will be documented.
      - N/A; Only caregivers of children ages 0-21 will be recruited and consented (electronically or with pen and paper) to participate in this study- no children will be enrolled.

**Cognitively Impaired Adults**

- - - Describe the process to determine whether an individual is capable of consent. The IRB allows the person obtaining assent to document assent on the consent document and does not routinely require assent documents and does not routinely require children to sign assent documents.
      - Study staff are trained to assess an individual’s capacity to provide written or electronic consent both prior to recruitment and during consent discussions. If study staff are unable to determine capacity to consent, the PI will be contacted securely to weigh in as an additional assessor.

**Adults Unable to Consent**

- - - List the individuals from whom permission will be obtained in order of priority. (E.g., durable power of attorney for health care, court appointed guardian for health care decisions, spouse, and adult child.)
      - For research conducted in the state, review “SOP: Legally Authorized Representatives, Children, and Guardians (HRP-013)” to be aware of which individuals in the state meet the definition of “legally authorized representative.”
      - For research conducted outside of the state, provide information that describes which individuals are authorized under applicable law to consent on behalf of a prospective subject to their participation in the procedure(s) involved in this research. One method of obtaining this information is to have a legal counsel or authority review your protocol along the definition of “legally authorized representative” in “SOP: Legally Authorized Representatives, Children, and Guardians (HRP-013).”

No adults unable to consent will be enrolled in the study

- - - Describe the process for assent of the subjects. Indicate whether:
      - Assent will be required of all, some, or none of the subjects. If some, indicated, which subjects will be required to assent and which will not.
      - If assent will not be obtained from some or all subjects, an explanation of why not.
      - Describe whether assent of the subjects will be documented and the process to document assent. The IRB allows the person obtaining assent to document assent on the consent document and does not routinely require assent documents and does not routinely require subjects to sign assent documents.

No adults unable to consent will be enrolled in the study

**Adults Unable to Consent**

- - - For HUD uses provide a description of how the patient will be informed of the potential risks and benefits of the HUD and any procedures associated with its use.
      - N/A

# Process to Document Consent in Writing

- 1. Describe whether you will be following “SOP: Written Documentation of Consent (HRP-091).” If not, describe whether and how consent of the subject will be documented in writing.
     - We will be following SOP: Written Documentation of Consent (HRP-091). Before the study, participants will be instructed to read, agree, and sign the document with paper and pen or electronically via an online electronic signature on REDCap, whichever they prefer.
  2. If your research presents no more than minimal risk of harm to subjects and involves no procedures for which written documentation of consent is normally required outside of the research context, the IRB will generally waive the requirement to obtain written documentation of consent.
     - N/A
  3. (If you will document consent in writing, attach a consent document. If you will obtain consent, but not document consent in writing, attach a consent script. Review “CHECKLIST: Waiver of Written Documentation of Consent (HRP-411)” to ensure that you have provided sufficient information. You may use “TEMPLATE CONSENT DOCUMENT (HRP-502)”to create the consent document or script.)
     - See attached documents.

# Setting

- 1. Describe the sites or locations where your research team will conduct the research.
     - Identify where your research team will identify and recruit potential subjects.
       - Children who received HSCT and their primary caregiver will be identified through weekly clinic schedules and caregivers will be recruited prior to their child’s discharge from the inpatient HSCT floor.
     - Identify where research procedures will be performed.
       - At Nationwide Children’s Hospital on the inpatient HSCT floor, upon enrollment and then weekly for 3 months via online REDCap survey administration. Surveys will be completed via REDCap text link, secure email, or in-person during clinic follow-up visits.
     - Describe the composition and involvement of any community advisory board.
       - N/A
     - For research conducted outside of the organization and its affiliates describe:
       - Site-specific regulations or customs affecting the research for research outside the organization.
         1. N/A
       - Local scientific and ethical review structure outside the organization.
         1. N/A

# Resources Available

- 1. Describe the resources available to conduct the research: For example, as appropriate:
     - Justify the feasibility of recruiting the required number of suitable subjects within the agreed recruitment period. For example, how many potential subjects do you have access to? What percentage of those potential subjects do you need to recruit?
       - Estimates of potential recruitment/retention rates are based on cancer registry data and strong documented recruitment experience at NCH. With approximately 80 transplants annually at NCH, we will have ample eligible families to approach. The goal is to enroll at least 50 families, with approximately 20% attrition prior to completion of all measures for a total of 50 (~80% of enrolled dyads). We anticipate 85% mothers as primary caregivers, and children participants will be equally distributed by sex.
     - Describe the time that you will devote to conducting and completing the research.
       - Study staff will continuously work on conducting and completing the study during the 3 years of the study.
     - Describe your facilities.
       - Nationwide Children’s Hospital is a large, Midwestern pediatric academic hospital.
     - Describe the availability of medical or psychological resources that subjects might need as a result of an anticipated consequences of the human research.
       - If a participant does become distressed, they will be offered a break and reminded that they can skip items or discontinue participation at any time. We also have an established protocol for managing risk for harm and adhere to mandated reporting requirements. When there is concern for harm (i.e., suicide or abuse) a structured follow-up is conducted by study staff and/or the PI to assess risk and need for a referral to child protective services by the PI. Any participant indicating a risk for self-harm will be referred to appropriate services based on immediacy of the threat. All hospitals have established social work and psychology crisis teams and services available
     - Describe your process to ensure that all persons assisting with the research are adequately informed about the protocol, the research procedures, and their duties and functions.
       - Because of the sensitive nature of the project, staff members have extensive training in research ethics and are supervised closely by the PI. The PI has extensive experience working with families exposed to the stress of severe childhood illnesses. Families will be informed of research procedures and expectations for participation

# Multi-Site Research*

- 1. *Study-Wide Number of Subjects**

*If this is a multicenter study, indicate the total number of subjects to be accrued across all sites.*

- - - N/A
  1. Study-Wide Recruitment Methods*
     - If this is a multicenter study and subjects will be recruited by methods not under the control of the local site (e.g., call centers, national advertisements) describe those methods. Local recruitment methods are described later in the protocol.
       - N/A
     - Describe when, where, and how potential subjects will be recruited.
       - N/A
     - Describe the methods that will be used to identify potential subjects.
       - N/A
     - Describe materials that will be used to recruit subjects. (Attach copies of these documents with the application. For advertisements, attach the final copy of printed advertisements. When advertisements are taped for broadcast, attach the final audio/video tape. You may submit the wording of the advertisement prior to taping to preclude re-taping because of inappropriate wording, provided the IRB reviews the final audio/video tape.)
       - N/A
     - If this is a multi-site study where you are the lead investigator, describe the processes to ensure communication among sites. See “WORKSHEET: Communication and Responsibilities (HRP-830).” All sites have the most current version of the protocol, consent document, and HIPAA authorization.
       - N/A
     - All required approvals (initial, continuing review and modifications) have been obtained at each site (including approval by the site’s IRB of record).
       - N/A
     - All modifications have been communicated to sites, and approved (including approval by the site’s IRB of record) before the modification is implemented.
       - N/A
     - All engaged participating sites will safeguard data, including secure transmission of data, as required by local information security policies.
       - N/A
     - All local site investigators conduct the study in accordance with applicable federal regulations and local laws.
       - N/A
     - All non-compliance with the study protocol or applicable requirements will reported in accordance with local policy.
       - N/A
  2. Describe the method for communicating to engaged participating sites (see “WORKSHEET: Communication and Responsibilities (HRP-830)”):
     - Problems (inclusive of reportable events).
       - N/A
     - Interim results.
       - N/A
     - The closure of a study
       - N/A
  3. If this is a multicenter study where you are a participating site/investigator, describe the local procedures for maintenance of confidentiality. (See “WORKSHEET: Communication and Responsibilities (HRP-830).”)
     - Where and how data or specimens will be stored locally?
       - N/A
     - How long the data or specimens will be stored locally?
       - N/A
     - Who will have access to the data or specimens locally?
       - N/A
     - Who is responsible for receipt or transmission of the data or specimens locally?
       - N/A
     - How data and specimens will be transported locally?
       - N/A

# ****27.0 Protected Health Information Recording****

1. **Indicate which subject identifiers will be recorded for this research.**

Name

Complete Address

Telephone or Fax Number

Social Security Number (do not check if only used for ClinCard)

Dates (treatment dates, birth date, date of death)

Email address , IP address or url

Medical Record Number or other account number

Health Plan Beneficiary Identification Number

Full face photographic images and/or any comparable images (x-rays)

Account Numbers

Certificate/License Numbers

Vehicle Identifiers and Serial Numbers (e.g. VINs, License Plate Numbers)

Device Identifiers and Serial Numbers

Biometric identifiers, including finger and voice prints

Other number, characteristic or code that could be used to identify an individual

None (Complete De-identification Certification Form)

**2.0  Check the appropriate category and attach the required form* on the Local Site Documents, #3. Other Documents, page of the application.  (Choose one.)**

Patient Authorization will be obtained. (Include the appropriate HIPAA language (see Section 14 of consent template) in the consent form OR attach the HRP-900, HIPAA AUTHORIZATION form.)

Protocol meets the criteria for waiver of authorization. (Attach the HRP-901, WAIVER OF HIPAA AUTHORIZATION REQUEST form.)

Protocol is using de-identified information. (Attach the HRP-902, DE-IDENTIFICATION CERTIFICATION form.) (Checked "None" in 1.0 above)

Protocol involves research on decedents. (Attach the HRP-903, RESEARCH ON DECEDENTS REQUEST form.)

Protocol is using a limited data set and data use agreement. (Contact the Office of Technology Commercialization to initiate a Limited Data Use Agreement.

***Find the HIPAA forms in the IRB Website Library, Templates.**

**Attach the appropriate HIPAA form on the “Local Site Documents, #3. Other Documents”, page of the application.**

1. **How long will identifying information on each participant be maintained?**

Identifiers are stored for 6 years after study closure to meet OHRP and HIPAA regulation

**Describe any plans to code identifiable information collected about each participant.** Each participant will be assigned a subject ID number which will be used to identify all study data. Identifying information will be stored separately from study data (e.g., consent forms, participant contact logs) and a recruitment spreadsheet used by staff will be the only place that subject numbers and identifiers will be linked. These will be password protected and stored only on secure servers.

1. **Check each box that describes steps that will be taken to safeguard the confidentiality of information collected for this research:**

X **Research records will be stored in a locked cabinet in a secure location**

X **Research records will be stored in a password-protected computer file**

X **The list linking the assigned code number to the individual subject will be maintained separately from the other research data**

X **Only certified research personnel will be given access to identifiable subject information**

1. **Describe the provisions included in the protocol to protect the privacy interests of subjects, where "privacy interests" refer to the interest of individuals in being left alone, limiting access to them, and limiting access to their information. (This is not the same provision to maintain the confidentiality of data.)**

See above regarding privacy protections.

**Confidential Health Information**

1. **Please mark all categories that reflect the nature of health information to be accessed and used as part of this research.**

Demographics (age, gender, educational level)

Diagnosis

Laboratory reports

Radiology reports

Discharge summaries

Procedures/Treatments received

Dates related to course of treatment (admission, surgery, discharge)

Billing information

Names of drugs and/or devices used as part of treatment

Location of treatment

Name of treatment provider

Surgical reports

Other information related to course of treatment

None

1. **Please discuss why it is necessary to access and review the health information noted in your response above.**

This information will be recorded to verify participant eligibility and to examine demographic (e.g., sex, age, income), medical (e.g., diagnosis, length of therapy, type of transplant) and family factors (e.g., family communication, barriers) in the prediction of level of adherence over time.

3.0 Is the health information to be accessed and reviewed the minimal necessary to achieve the goals of this research?  Yes  No

4.0 Will it be necessary to record information of a sensitive nature?  Yes  No

5.0 Do you plan to obtain a federally-issued Certificate of Confidentiality as a means of protecting the confidentiality of the information collected?  Yes  No
